# Novel role of L-2-HG in regulating HIF1A signaling pathway and iron death resistance in renal cancer brain metastasis

**DOI:** 10.1038/s41419-025-08068-z

**Published:** 2025-11-06

**Authors:** Guangxiang Liu, Shengjie Zhang, Haixiang Qin, Kuiqiang He, Renjie Li, Hongqian Guo

**Affiliations:** Department of Urology, Nanjing Drum Tower Hospital, The Affiliated Hospital of Nanjing University Medical School; Institute of Urology, Nanjing University, Nanjing, PR China

**Keywords:** Renal cell carcinoma, Renal cell carcinoma

## Abstract

L-2-hydroxyglutarate (L-2-HG) functions as a metabolite implicated in the progression of various tumors. HIF1A, a central regulator of the hypoxic response, is known to be regulated by several metabolites. This study aims to elucidate whether L-2-HG regulates the function of HIF1A through histone lactylation modification, thereby contributing to brain metastasis in renal cell carcinoma (RCC). A mouse model of RCC brain metastasis was constructed, and high-throughput metabolomics, transcriptomics, and proteomics sequencing analyses were conducted. Bioinformatics analysis revealed that L-2-HG enhanced HIF1A expression by promoting histone lactylation modification, which suppressed ferroptosis and facilitated RCC brain metastasis. In vitro cellular experiments were conducted, including cell treatment, transfection, chromatin immunoprecipitation (ChIP), malignant phenotype detection assays, Western blotting, and RT-qPCR. The results showed that L-2-HG increased the lactylation modification of HIF1A and enhanced the resistance of renal cancer cells to ferroptosis, thereby increasing cell proliferation, migration, and invasion. In vivo experiments using a nude mouse lung metastasis model demonstrated the mechanism through which L-2-HG promoted RCC brain metastasis.

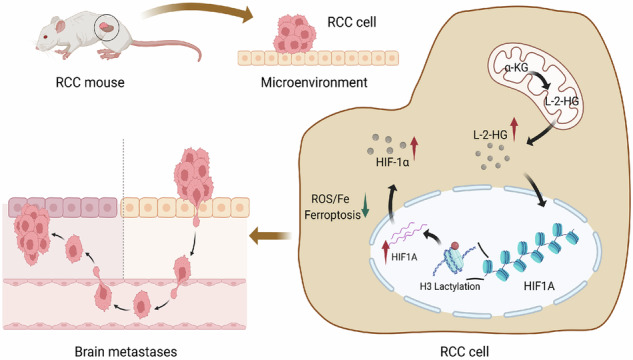

## Introduction

Renal cell carcinoma (RCC) is among the most common malignant tumors worldwide, and its highly invasive and metastatic nature severely impacts patient survival and quality of life [1–3]. Brain metastasis represents a major cause of disease progression in RCC, but its mechanism remains unclear [4, 5]. L-2-hydroxyglutarate (L-2-HG), as a metabolic product, has been identified as a key regulatory factor in the progression of various tumors [6]. HIF1A is a core hypoxia-inducible transcription factor, and its function and metabolic role in tumor regulation continue to be a hot topic of research [7–9]. Therefore, this study aimed to investigate whether L-2-HG regulates the function of HIF1A through histone lactylation modification, thereby influencing the occurrence and development of RCC brain metastasis.

L-2-HG, as a metabolic product, has been proven to play an important regulatory role in various tumors [10, 11]. However, research on the interaction between L-2-HG and HIF1A is relatively limited. HIF1A, as a core hypoxia-inducible transcription factor, has gained extensive attention for its role in tumor initiation and progression [12–15]. Therefore, a deeper understanding of the relationship between L-2-HG and HIF1A is of great significance for revealing the molecular mechanisms of RCC brain metastasis.

In this study, an animal model of RCC brain metastasis was used to simulate the process of RCC metastasis in the brain. In vivo fluorescence imaging technology was employed to monitor tumor formation and metastasis. By conducting high-throughput transcriptome, metabolome, and proteome sequencing analysis on metastatic tissues and control tissues, differential genes, metabolites, and proteins associated with RCC brain metastasis were identified [16–18]. Integrated analysis of multi-omics data was conducted to investigate the molecular mechanisms of L-2-HG and HIF1A in the development of RCC brain metastasis.

The objective of this study is to elucidate whether L-2-HG regulates the occurrence of RCC brain metastasis by promoting lactylation modification of HIF1A. Effects of L-2-HG on HIF1A lactylation were assessed through in vitro cellular experiments and in vivo mouse models. Additional analyses evaluated its influence on RCC cell proliferation, migration, invasion, and ferroptosis. A pulmonary metastasis model in nude mice was further used to validate the role and mechanisms of L-2-HG in RCC metastasis.

The study is expected to demonstrate L-2-HG promotes RCC brain metastasis by enhancing lactylation modification of HIF1A. Results may help elucidate the functional significance of L-2-HG and HIF1A in RCC brain metastasis and provide new targets for its treatment. The findings have the potential to improve the treatment outcomes of RCC metastasis patients, offering more precise and effective therapeutic strategies and providing scientific evidence for clinical practice.

## Results

### Mouse model and pathophysiological characteristics of brain metastasis in RCC

**Fig. 1:**
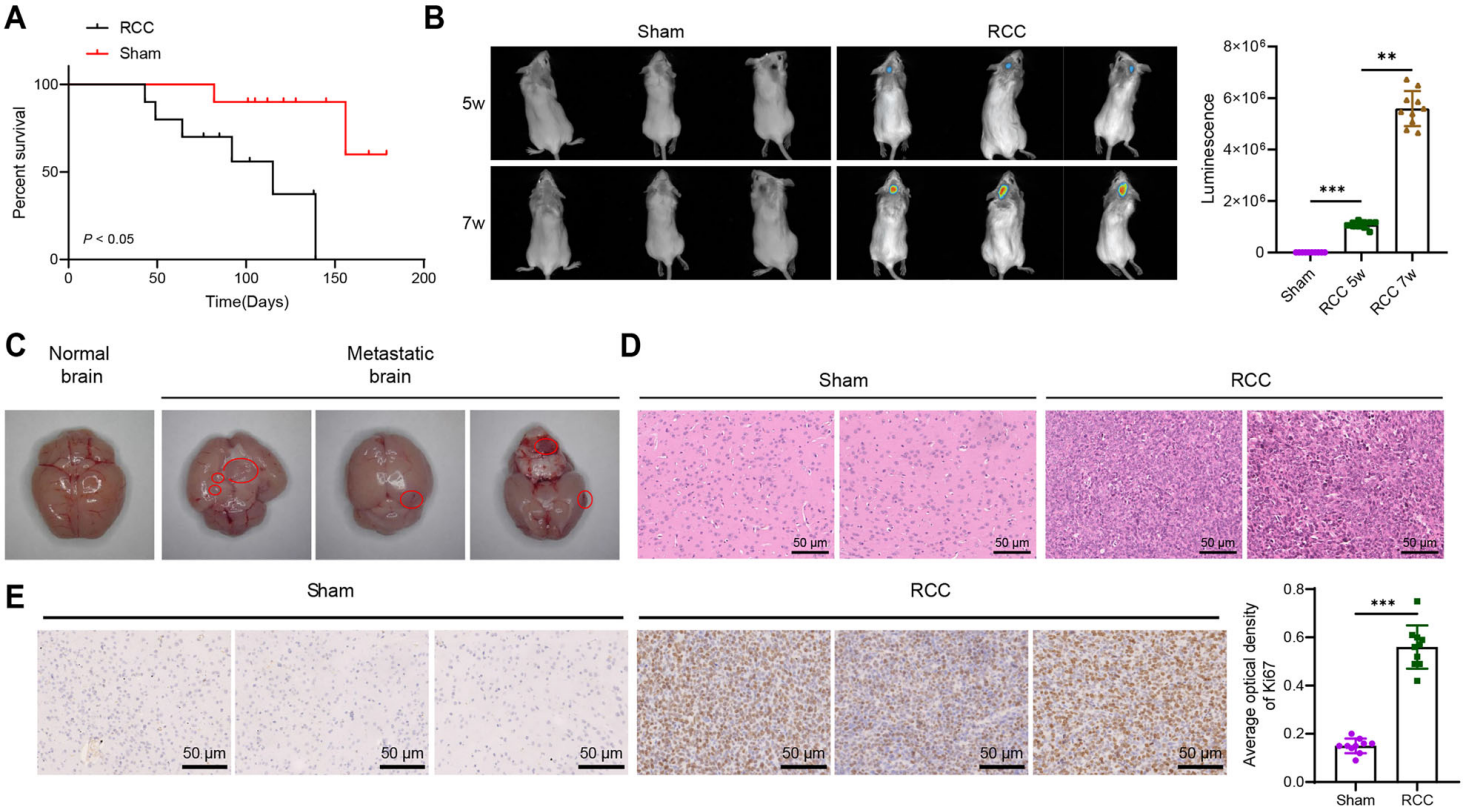
Construction and validation of the mouse model of RCC brain metastasis. **A** Kaplan–Meier survival curves of mice in the Sham and RCC groups; **B** In vivo fluorescence imaging to detect the growth rate and volume of intracranial tumors, with fluorescence intensity statistics on the right; **C** Representative images of brain tissue and metastatic lesions following euthanasia; **D** Morphology of brain tissue and tumor cells in the Sham and RCC groups observed by H&E staining, scale bar = 50 μm; **E** Expression levels of cell proliferation-related factor Ki67 detected by immunohistochemistry, scale bar: 50 μm. Each group consisted of *N* = 10 mice, where the symbols ** and *** represent *P* < 0.01 and *P* < 0.001, respectively, indicating significant differences between the two groups.

Brain metastasis is a poor prognostic feature of metastatic RCC. To accurately replicate the pathophysiological process of RCC brain metastasis in vivo, we established a mouse model of RCC brain metastasis through intra-carotid artery injection [19–21]. In vitro fluorescence imaging confirmed a positive correlation between luminescence intensity and cell number, indicating successful transfection with the luciferase gene and reliable visualization of cells at high sensitivity (Fig. S1). Following intra-carotid artery injection of luciferase-expressing RCC cells, mice in the RCC group developed behavioral abnormalities, including rapid weight loss, hunchback posture, impaired feeding and drinking, unilateral or bilateral limb dysfunction, and immobility. Based on these observations, euthanasia was performed, and survival time was calculated. The RCC group exhibited a median survival of 88 days, significantly shorter than that of the Sham group (Fig. 1A). In vivo fluorescence imaging showed the presence of metastatic lesions in different brain regions of mice in the RCC group, with signal intensity increasing over time. No such fluorescence signals were observed in the control group (Fig. 1B).

Hematoxylin and eosin (H&E) staining and immunohistochemistry were subsequently performed on mouse brain tissues. Macroscopically visible metastatic lesions were observed in euthanized mice, distributed across various regions, including the cortex and ventricles (Fig. 1C). H&E staining revealed enlarged tumor cells, an increased nuclear-to-cytoplasmic ratio, active mitotic figures, and prominent nucleoli in the RCC group (Fig. 1D). Moreover, immunohistochemical detection of the cell proliferation marker Ki67 demonstrated a significant increase in Ki67 content in the brain tissues of the RCC group (Fig. 1E). These findings confirmed the successful formation of tumors in the brain by RCC cells, characterized by typical metastatic features, and validated the utility of the model for subsequent experimental investigations.

### Analysis of changes in energy metabolism pathways and associated metabolites during brain metastasis of RCC

**Fig. 2:**
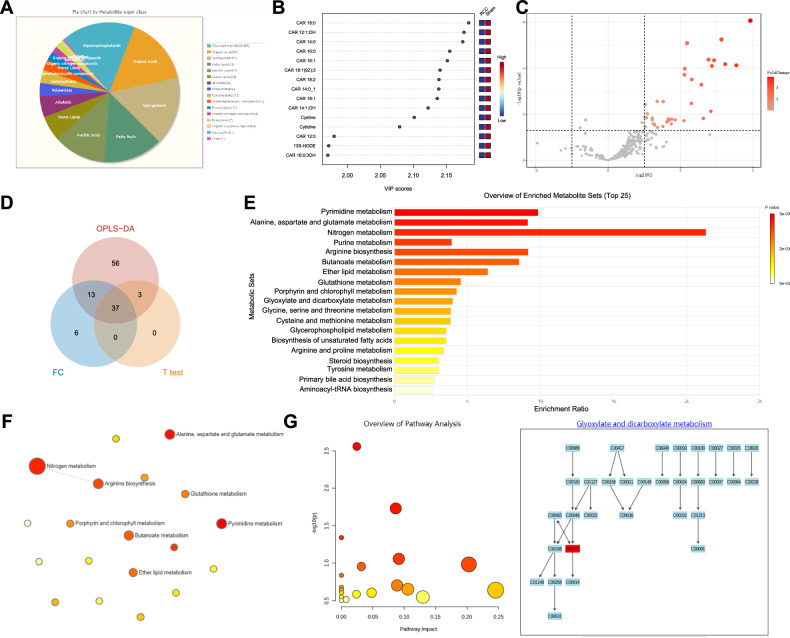
Analysis of metabolomics data reveals key metabolites in RCC brain metastasis. **A** Pie chart of metabolite composition in mouse brain tissue classified by “Superclass”; **B** VIP values of the top-ranked metabolites analyzed by OPLS-DA; **C** Volcano plot of differentially regulated metabolites based on Fold Change analysis and *T* test; **D** Venn diagram showing the intersection of differentially regulated metabolites obtained from OPLS-DA analysis (VIP > 1), Fold Change analysis (|logFC| > 2), and *T* test (*P* < 0.05); **E** Bar plot of functional enrichment analysis results for differentially regulated metabolites in the MetaboAnalyst database; **F** Network diagram of pathway enrichment results; node size indicates the number of mapped metabolites, and color intensity indicates enrichment significance; **G** Pathway enrichment analysis results for differentially regulated metabolites in the MetaboAnalyst database (circle size and color depth have the same meaning as in the previous figure), demonstrating the “Glyoxylate and dicarboxylate metabolism” pathway and enriched metabolites. Each group consisted of *N* = 4 samples.

Previous studies have established close associations between tumor development and alterations in energy metabolism pathways, particularly the tricarboxylic acid cycle and glycolysis [22–25]. Therefore, a high-throughput metabolomics analysis was conducted on brain tissue samples from mice with RCC brain metastases and control mice. Data correction and normalization were performed to enable subsequent statistical analysis (Fig. S2A), followed by an assessment of metabolite composition. The results indicated that metabolites associated with energy metabolism, such as Glycerophospholipids, Organic acids, and Fatty Acyls, had higher proportions among all metabolites (Figs. 2A and S2B).

Orthogonal partial least squares discriminant analysis (OPLS-DA) was employed to identify metabolites related to brain metastasis of RCC. OPLS-DA effectively minimized both within-group variability and unrelated random error while addressing overfitting. The score plot demonstrated clear separation between the brain metastasis and control groups (Fig. S2C). Based on the variable importance projection (VIP) values obtained from the OPLS-DA model (Fig. S2D), metabolites with VIP scores greater than 1 were selected for further analysis. The top 15 metabolites ranked by VIP values are shown in Fig. 2B, along with the correlation results among different metabolites (Fig. S2E). Further integration of Fold Change (|logFC| > 2, Fig. S2F) and *T*-test (*P* < 0.05, Fig. S2G) led to the identification of 37 differential metabolites (including L-2-Hydroxyglutaric acid) (Fig. 2C, D and Table S1).

To investigate the major impact pathways of these differential metabolites on brain metastasis of RCC, we performed metabolic pathway enrichment analysis using the MetaboAnalyst website. The results revealed that the most significantly enriched pathways were primarily involved in lipid metabolism (such as “Glycerophospholipid metabolism” and “Biosynthesis of unsaturated fatty acids”) and amino acid synthesis and degradation (including “Alanine, aspartate and glutamate metabolism,” “Nitrogen metabolism,” and “Arginine biosynthesis”) (Figs. 2E, F and S3). Additionally, the various organic acid intermediates generated by these metabolic pathways were further oxidatively decomposed through energy metabolism-related pathways, such as “Glyoxylate and dicarboxylate metabolism” (Fig. 2G). These alterations of metabolic pathways are highly relevant to the occurrence and development of various diseases, including tumors [26]. Further analysis focused on the top 10 differential metabolites (ranked by |logFC|), which were enriched in key metabolic pathways. Among them, L-2-HG was identified as strongly associated with tumor-related pathways, including glycolysis and lactate metabolism. Lactate functions not only as a metabolic byproduct but also as a regulator of epigenetic modifications and gene expression. L-2-HG, as a tumor metabolic biomarker, has been shown to accumulate in tumor cells and is closely linked to the development of various cancers [27]. Furthermore, it has been shown to modulate both adaptive and innate immunity [28], thereby influencing the tumor microenvironment. Recent studies have indicated that L-2-HG can promote tumor progression through the induction of aberrant methylation, lactylation, and hypoxia-related reactions [27, 29, 30]. Based on these findings, we hypothesize that L-2-HG may contribute to the development of brain metastasis of RCC by inducing abnormal histone lactylation modifications.

### The impact and mechanism of L-2-HG-Mediated histone modifications on brain metastasis in RCC: research findings

**Fig. 3:**
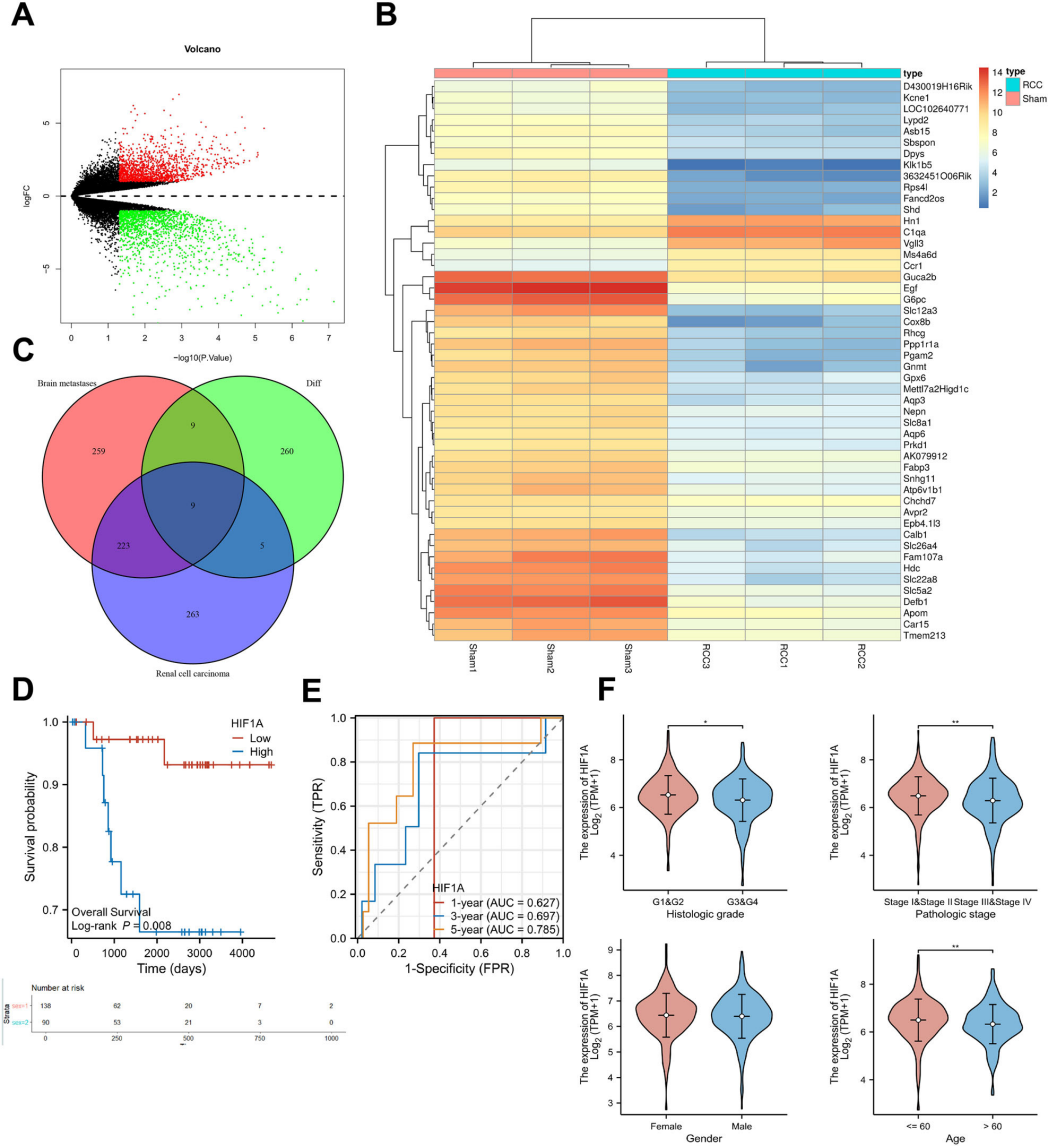
Analysis of transcriptomics data reveals key genes involved in RCC brain metastasis. **A** Volcano plot of differentially expressed genes (DEGs) from RNA-seq analysis (red and green dots represent upregulated and downregulated DEGs in the RCC group, respectively, with each group consisting of *N* = 3 samples); **B** Heatmap of expression levels of the most significantly upregulated differentially expressed genes in the RNA-seq analysis results, with each group consisting of *N* = 3 samples; **C** Venn diagram depicting the intersection of upregulated differentially expressed genes from the RNA-seq analysis and genes related to “Renal cell carcinoma” and “Brain metastases” in the GeneCards database; **D** Survival analysis of HIF1A expression in RCC-related gene expression data from the TCGA database, where the blue and red lines represent the high-expression group and low-expression group of HIF1A, respectively (*N* = 45 per group); **E** ROC curve showing the predictive performance of 1, 3, and 5-year survival rates of 90 RCC patients based on the expression level of HIF1A in the TCGA renal cell carcinoma dataset, with the area under the curve representing the prediction success rate; **F** Correlation analysis between the expression level of HIF1A and clinical indicators such as tumor grade, stage, age, and gender, where the symbols * and ** represent *P* < 0.05 and *P* < 0.01, respectively.

To further elucidate the effects of L-2-HG-mediated histone modifications on target genes or proteins, as well as the mechanisms in promoting brain metastasis in RCC, we conducted transcriptomic and proteomic sequencing on brain tissue samples from both RCC and Sham mouse groups. RNA sequencing identified 701 differentially expressed genes (DEGs) in brain tissues from the RCC group compared to controls, including 283 upregulated and 418 downregulated genes (Fig. 3A). Histone lactylation at lysine residues has been reported to directly enhance gene expression [31]. Based on this evidence, analysis focused on the upregulated genes. The expression profiles of the top 50 significantly upregulated genes were visualized (Fig. 3B). To identify genes relevant to RCC brain metastasis, keyword searches for “renal cell carcinoma” and “brain metastases” were conducted in the GeneCards database. The top 500 genes, ranked by relevance score, were selected as key disease-associated genes. An intersection between these genes and the upregulated DEGs yielded nine key genes associated with RCC brain metastasis: KRAS, MYC, BRCA1, HIF1A, VIM, NRG1, PVT1, BIRC5, and CDKN1A (Fig. 3C).

Furthermore, we performed additional filtering of the above-mentioned nine genes using the TCGA database. The pairwise differential analysis of RCC samples showed no statistically significant differences in expression between KRAS and PVT1 between tumor tissues and matched adjacent normal tissues (Fig. S4A). Then, clinical correlation analyses were conducted on the remaining seven genes. Based on the median gene expression levels in RCC samples, patients were divided into high-expression and low-expression groups for survival analysis. Significant differences in overall survival were observed between these groups for BRCA1, NRG1, BIRC5, and HIF1A (Figs. 3D and S4B). Given the established role of histone modifications in target gene expression and tumor progression, patients in the low-expression group have higher survival rates. Hence, BRCA1, BIRC5, and HIF1A aligned with this pattern. Additionally, receiver operating characteristic (ROC) curve analysis was used to evaluate the prognostic accuracy of these genes (Figs. 3E and S4C). HIF1A demonstrated strong correlations with multiple clinical indicators (Fig. 3F), suggesting its critical role in the occurrence and development of various tumors, particularly RCC. Therefore, we postulate that HIF1A may be one of the target genes regulated by L-2-HG-mediated histone modifications, and its upregulation promotes the development of brain metastasis in RCC.

### Mechanisms and functional enrichment analysis of HIF1A-mediated RCC Brain metastasis

**Fig. 4:**
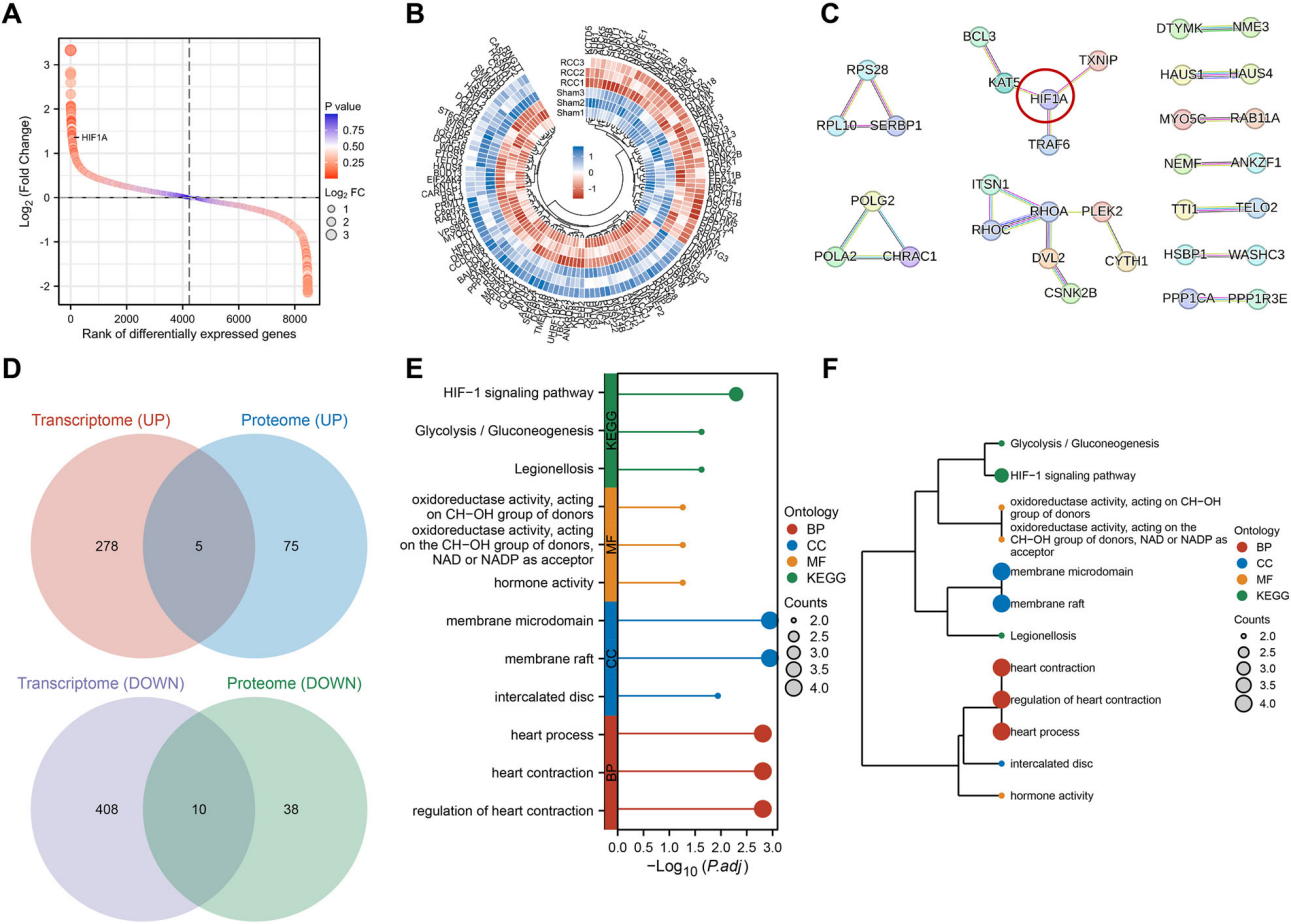
Molecular mechanisms of HIF1A’s impact on RCC brain metastasis explored through multi-omics integration analysis. **A** Differential protein sorting plot for proteomic sequencing, *N* = 3 per group; **B** Circular heat map illustrating the most significant differentially expressed proteins, *N* = 3 per group; **C** PPI protein interaction network analysis of differentially expressed proteins (Created with BioRender.com); **D** Venn diagram identifying overlapping upregulated and downregulated genes/proteins from transcriptomic and proteomic datasets. Shared upregulated genes/proteins included TLR2, CXADR, EDN1, HOXB5, and HIF1A; shared downregulated molecules included JMJD6, KCNAB1, LDHB, BGN, PROKR1, ADM, ENO1, BNIP3, CAVIN2, and DSC2; **E** Lollipop plot of GO and KEGG enrichment results for intersecting genes/proteins; **F** Clustered dendrogram of GO and KEGG enrichment results for intersecting genes/proteins.

To validate the transcriptomic findings and investigate the mechanisms underlying HIF1A-mediated RCC brain metastasis, we performed proteomic sequencing and in-depth analysis of brain tissue from RCC and Sham mice. Tandem mass tag (TMT)-labeled quantitative proteomics technology was employed for protein profiling. A total of 9964 proteins were identified, among which 128 showed significant differential expression, including 80 upregulated and 48 downregulated proteins (Fig. 4A, B). Among the genes associated with the prognosis of TCGA RCC patients, including BRCA1, BIRC5, and HIF1A, only HIF1A exhibited upregulation in the brain tissue of RCC brain metastasis mice (Fig. 4A). To validate the central role of HIF1A in the occurrence of RCC brain metastasis, a protein-protein interaction (PPI) analysis was performed using the STRING database based on the 128 differentially expressed proteins. The PPI network revealed that HIF1A and RHOA occupied central positions within the network (Fig. 4C). These findings, combined with previous metabolomics and transcriptomics data analysis results, further confirmed the critical role of HIF1A in the occurrence of RCC brain metastasis.

An integrated analysis of transcriptomic and proteomic datasets was subsequently performed to identify intersecting upregulated and downregulated genes/proteins. A total of 15 overlapping genes/proteins were identified, including five upregulated (including HIF1A) and ten downregulated (Fig. 4D). Gene Ontology (GO) and Kyoto Encyclopedia of Genes and Genomes (KEGG) enrichment analyses revealed these molecules were primarily involved in the HIF-1 signaling pathway, legionellosis, and glycolysis/gluconeogenesis (Fig. 4E, F), highlighting the concordance between transcriptomic and proteomic profiles. In conjunction with the previously enriched pathways from metabolomics, we speculate that HIF1A-mediated tumor metastasis may be closely related to its regulation of tumor cell iron death. Recent studies have shown that HIF-1α is involved in the regulation of iron death in various tumors, further impacting the occurrence and development of tumors [32–34], which strongly supports our hypothesis.

Based on bioinformatics analysis and literature review, we hypothesize that L-2-HG may promote HIF1A by regulating of histone lactylation modification and facilitate RCC brain metastasis by affecting tumor cell iron death.

### L-2-HG facilitates HIF1A expression by regulating histone lactylation modification

**Fig. 5:**
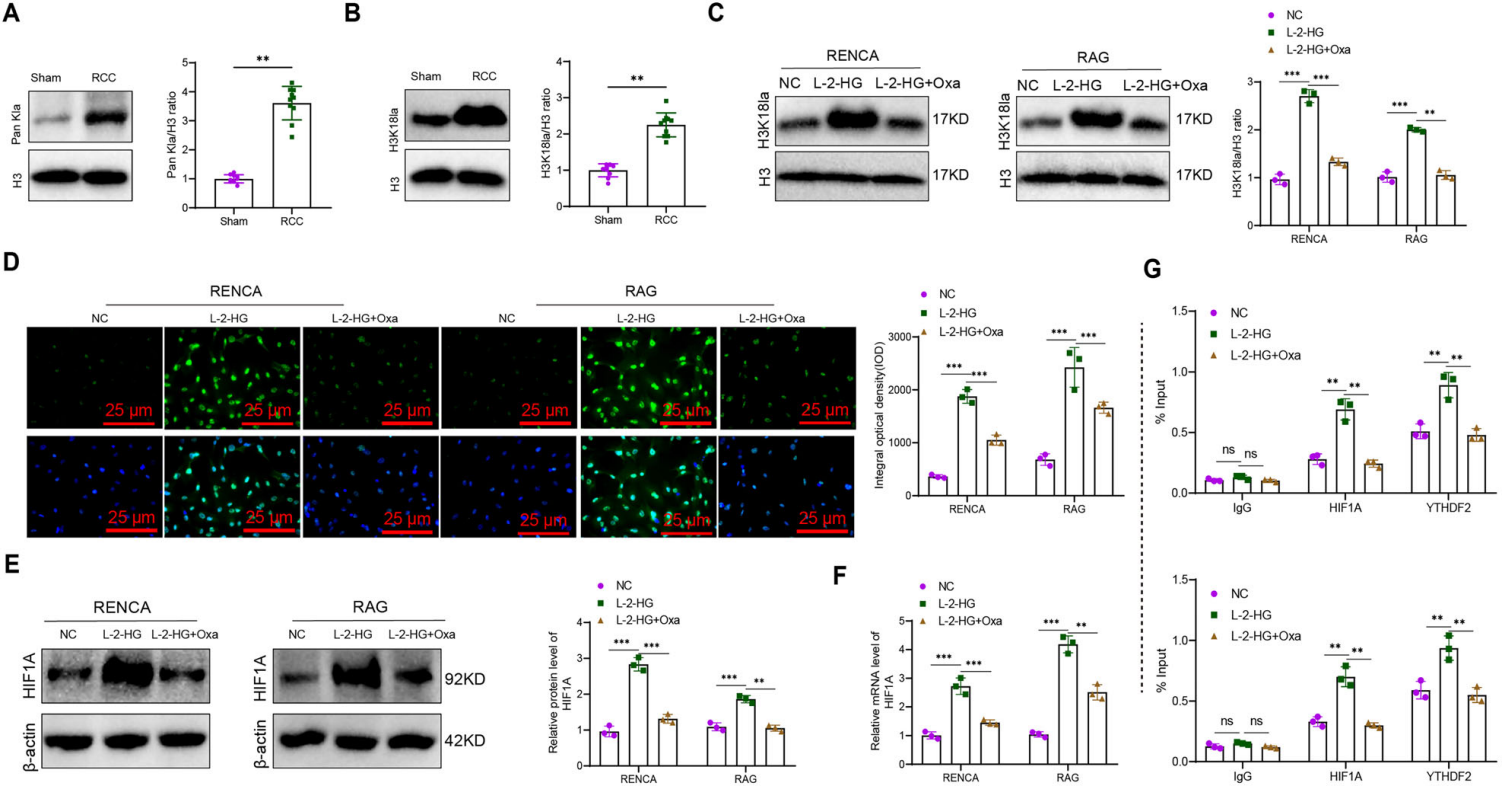
In vitro experimental validation of L-2-HG’s impact on HIF1A expression through histone lactylation modification. **A** Western blot analysis of overall lactylation (Pan Kla) levels in RCC and Sham groups (*N* = 3 per group); **B** Western blot analysis of lactylation modification (H3K18la) at the H3K18 site in RCC and Sham groups (*N* = 3 per group); **C** Western blot analysis of H3K18la levels in RENCA and RAG RCC cells under different treatment; **D** Immunofluorescence analysis of H3K18la levels in RENCA and RAG RCC cells treated through different methods, scale bar = 50 μm; **E** Western blot analysis of HIF1A protein expression in treated RENCA and RAG RCC; **F** RT-qPCR analysis of HIF1A mRNA levels in treated RENCA and RAG RCC cells; **G** ChIP-qPCR analysis of H3K18la levels in the HIF1A promoter region. IgG was used as a negative control and YTHDF2 served as the positive control. * and ** indicate *P* < 0.01 and *P* < 0.001, respectively, for comparison between the groups. The cell experiments were repeated three times.

Comprehensive multi-omic analysis of transcriptomic, metabolomic, and proteomic data indicated that L-2-HG may promote HIF1A expression by regulating histone lactylation modification. To validate this hypothesis, Western blot analysis was conducted to assess the global histone lactylation level in the brain tissues of Sham and RCC mice. The results demonstrated a significantly higher overall lactylation level (Pan Kla) in the RCC group compared to the Sham group, with lactylated histones predominantly located in the vicinity of histone H3 (Fig. 5A). These findings suggested that lactylation modification of histone H3 may be the main cause of the increased overall lactylation level in the RCC group. This result is consistent with previous studies showing that histone lactylation, particularly at the H3K18 residue (H3K18la), facilitates tumor progression [31]. To validate this observation, H3K18la levels were further examined and found to be significantly higher in the RCC group compared to the Sham group (Fig. 5B). The results indicated a positive association between elevated histone lactylation levels and RCC brain metastasis.

RENCA and RAG renal carcinoma cells were treated with L-2-HG to investigate its impact on histone lactylation modification and HIF1A expression. Previous research has shown significant H3K18la enrichment at the YTHDF2 promoter [35], and YTHDF2 was used as a positive control. Western blot analysis showed a significant increase in H3K18la levels in L-2-HG-treated RENCA and RAG cells (Fig. 5C). Additionally, oxalate, a sodium salt of oxalic acid, was used to control cellular lactate levels and inhibit histone lactylation modifications. The results revealed that oxalate treatment suppressed L-2-HG-mediated H3K18la (Fig. 5C). Similarly, immunofluorescence detection further confirmed the levels of H3K18la in each group (Fig. 5D). Western blot and RT-qPCR analyses indicated that L-2-HG treatment significantly upregulated HIF1A expression, while oxalate-mediated inhibition of lactylation reduced HIF1A levels (Fig. 5E, F). To assess whether H3K18la was enriched at the HIF1A promoter, ChIP-qPCR was performed. The results showed that L-2-HG promoted H3K18la levels in the HIF1A promoter region, an effect reversed by oxalate treatment (Fig. 5G). Notably, the expression trends of HIF1A mirrored those of the positive control YTHDF2 under different treatment conditions (Fig. 5G), supporting the conclusion that L-2-HG enhances H3K18la, while oxalate inhibits this modification.

Collectively, the findings demonstrated that L-2-HG facilitates HIF1A expression by regulating histone lactylation modification.

### L-2-HG promotes the proliferation, migration, and invasion of RCC cells while reducing ferroptosis susceptibility

**Fig. 6:**
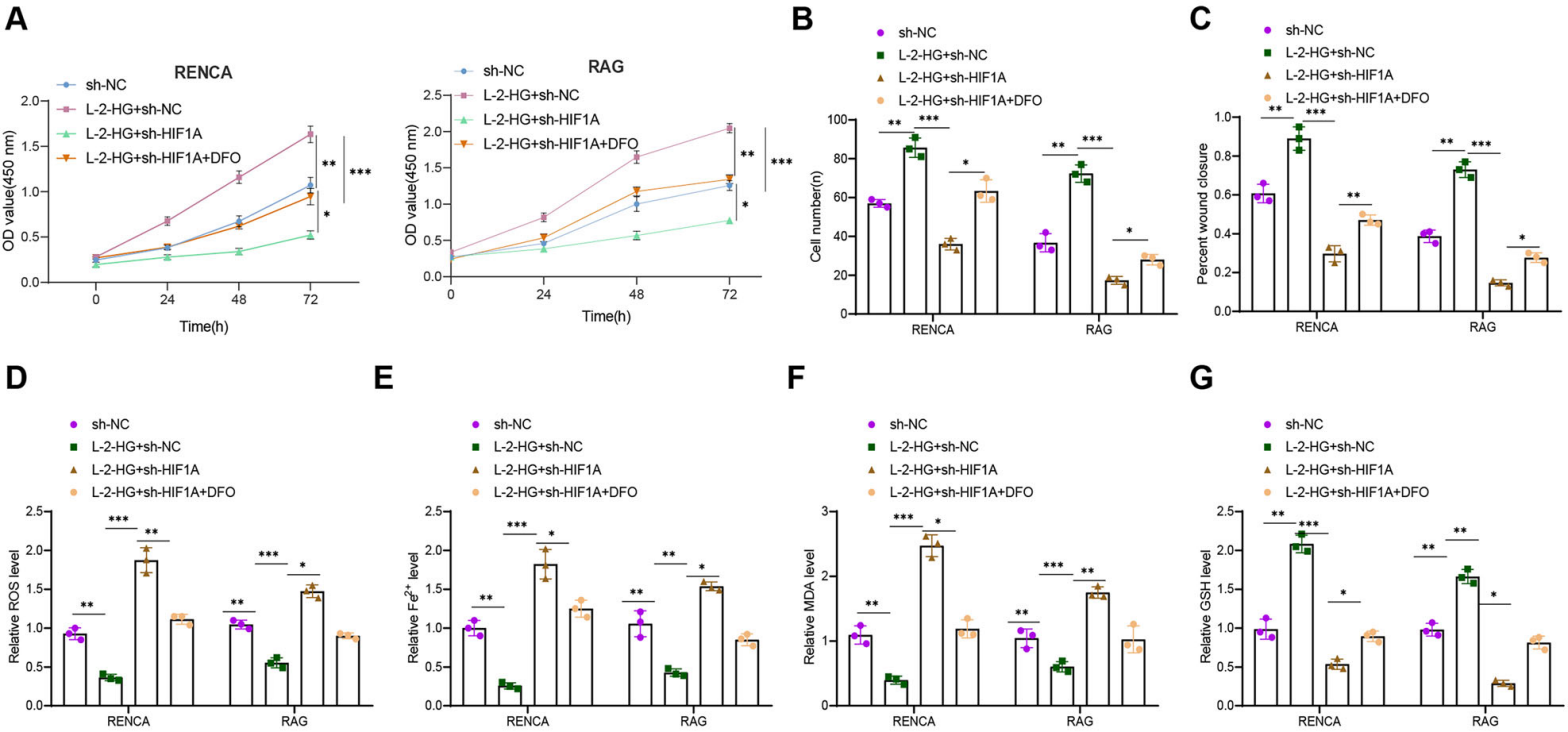
Impact of L-2-HG-regulated HIF1A expression on RCC cell ferroptosis and malignant phenotype. **A** CCK-8 assay was used to evaluate the proliferation ability of cells in each group; **B** Transwell assay for quantification of invasion ability of cells in each group; **C** Scratch assay for quantification of migration ability of cells in each group; **D** Detection of relative ROS levels in cells of each group; **E** Detection of relative Fe^2+^ levels in cells of each group; **F** Detection of relative MDA levels in cells of each group; **G** Detection of relative GSH levels in cells of each group, *, and *** indicate *P* < 0.05, *P* < 0.01, and *P* < 0.001, respectively, for comparison between the groups. The cell experiments were repeated three times.

To investigate how L-2-HG influences the development of brain metastasis in RCC by promoting HIF1A expression, we performed targeted transfections to knockdown HIF1A expression in cells (Fig. S5A). RT-qPCR analysis confirmed that sh-HIF1A-1 produced a more pronounced silencing effect than sh-HIF1A-2 in both RENCA and RAG cell lines. Therefore, sh-HIF1A-1 was selected as the silencing group (sh-HIF1A) for further experiments (Fig. S5B). Additionally, L-2-HG significantly increased the expression levels of HIF1A in sh-NC-transfected cells, while the L-2-HG+sh-HIF1A group exhibited significantly lower expression levels of HIF1A compared to the L-2-HG+sh-NC group (Fig. S5C). Subsequently, we further evaluated the impact of this regulatory mechanism on the malignant phenotype of tumor cells using in vitro cell experiments, including CCK-8 proliferation assay, scratch assay, and Transwell assay. The CCK-8 assay revealed that L-2-HG treatment significantly enhanced the proliferation of RENCA and RAG cells, while HIF1A knockdown attenuated this effect and reduced proliferation capacity (Fig. 6A).

Transwell assays were used to assess cell invasion and migration capabilities. The results demonstrated a significant increase in the invasion capability of RCC cells treated with L-2-HG, while the invasion capability of RCC cells with HIF1A knockdown was suppressed (Figs. 6B and S6A). Similarly, scratch assays showed consistent regulatory trends in terms of cell migration ability (Figs. 6C and S6B).

To further validate that L-2-HG promotes HIF1A expression and inhibits ferroptosis to promote the malignant phenotype of tumor cells, cells were treated with the ferroptosis inhibitor deferoxamine (DFO) during HIF1A knockdown. Consistent with our hypothesis, co-treatment with DFO and HIF1A knockdown significantly enhanced the proliferation and migration abilities of tumor cells (Figs. 6A–C and S6A, B). Intracellular levels of reactive oxygen species (ROS), glutathione (GSH), malondialdehyde (MDA), and Fe²⁺ were subsequently measured to evaluate ferroptosis. The results showed that after treatment with L-2-HG, the levels of ROS, MDA, and Fe^2+^ in the cells significantly decreased, while the level of GSH significantly increased, indicating inhibition of ferroptosis. Compared to tumor cells treated with L-2-HG alone, HIF1A knockdown increased ferroptotic activity. In contrast, co-treatment with DFO and HIF1A knockdown reversed these effects, as evidenced by decreased ROS, MDA, and Fe²⁺ levels and elevated GSH levels relative to the L-2-HG+sh-HIF1A group (Fig. 6D–G). These results further confirmed that L-2-HG promotes HIF1A expression, inhibiting iron death in tumor cells.

In summary, our results demonstrated that L-2-HG inhibited iron death in tumor cells by promoting HIF1A expression and further promoted the malignant phenotype of RCC cells.

### Biological effects and mechanisms of L-2-HG promoting intra-tumoral metastasis of RCC

**Fig. 7:**
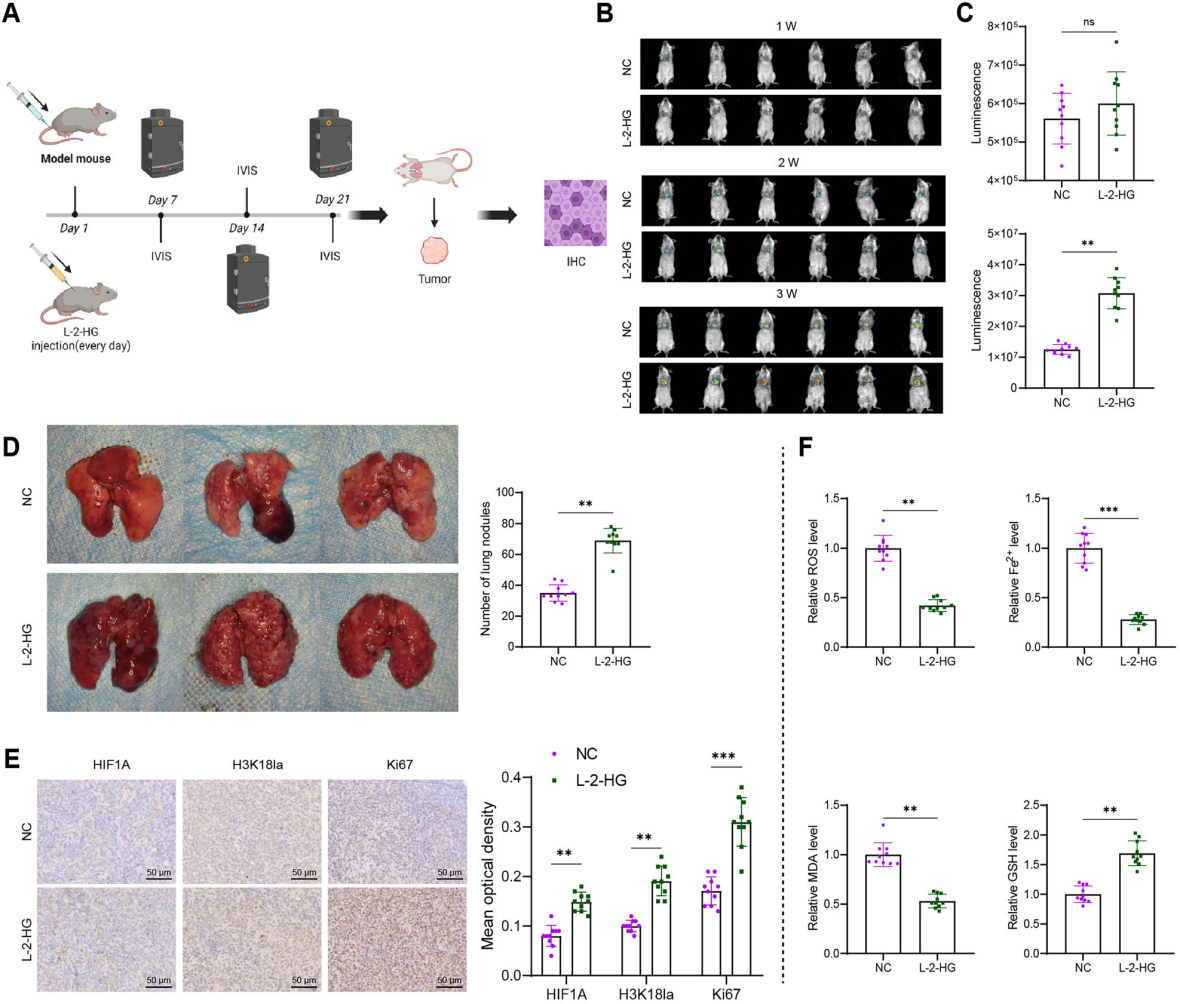
Analysis of L-2-HG’s impact on RCC cell metastatic ability in vivo. **A** Flowchart of in vivo experiments (Created with BioRender.com); **B** In vivo fluorescence imaging showing RCC cell metastasis progression; **C** Quantitative results of fluorescence intensity in vivo fluorescence imaging; **D** Images of tumor tissues and statistical results of lung metastatic nodules in a mouse lung metastasis model; **E** Immunohistochemical staining for HIF1A, H3K18la, and Ki67 in tumor tissues; scale bar = 50 μm; **F** Quantification of ferroptosis markers (ROS, Fe²⁺, MDA, and GSH) in tumor tissues. ns, *, and *** indicate no statistical difference, *P* < 0.05, and *P* < 0.001, respectively. Each group consisted of *N* = 10 mice.

In the final experiment, a lung metastasis model of RCC (RENCA) was established in nude mice via intravenous injection of fluorescent-labeled cells. To evaluate the in vivo biological effects of L-2-HG, intraperitoneal injection was administered (Fig. 7A). Live fluorescence imaging was performed for three consecutive weeks to monitor pulmonary metastasis. In the first week, no significant difference in fluorescence intensity was observed between the NC group and the L-2-HG group mice. However, in the second week, one mouse in the L-2-HG group showed a significant increase in fluorescence intensity compared to the other mice. By the third week, the fluorescence intensity of the L-2-HG group mice was significantly higher than that of the NC group mice (Fig. 7B, C). At the end of the observation period, isolated tumor tissues revealed larger and more numerous metastatic lung nodules in the L-2-HG group (Fig. 7D). These results further confirmed that L-2-HG promoted the metastatic capacity of RCC cells in vivo.

Next, immunohistochemistry was performed to assess the expression levels of relevant factors. The results showed a significant increase in HIF1A expression and H3K18la levels in the tumor tissues of the L-2-HG group mice. Additionally, Ki67 staining revealed a significantly higher proliferation ability of RCC cells in the L-2-HG group compared to the NC group (Fig. 7E). Furthermore, the levels of ROS, GSH, MDA, and Fe^2+^ were measured in the tumor tissues. The results indicated that ferroptosis was markedly inhibited in the L-2-HG group (Fig. 7F).

Collectively, the data supported the hypothesis that L-2-HG enhanced the metastatic ability of RCC cells in vivo by modulating histone lactylation modification, thereby upregulating HIF1A expression.

## Discussion

L-2-HG, as a metabolite, plays a crucial role in the progression of various tumors [6, 36, 37]. HIF1A, a key regulator of the hypoxic response, has been extensively studied for its metabolic role [13–15, 38]. The present study aimed to investigate whether L-2-HG regulates the function of HIF1A through histone lactylation modification, thereby influencing the brain metastasis of RCC. The objective was to elucidate a novel mechanism by which L-2-HG regulates the brain metastasis of RCC and to identify potential therapeutic targets.

Compared with previous approaches, the study incorporated several methodological innovations. Luciferase-expressing RCC cells were injected into the carotid artery to establish a brain metastasis model and real-time monitoring of tumor formation and metastasis was performed using an in vivo fluorescence imaging technique [39]. This technique allowed for more accurate and reliable observation of tumor dynamics and provided direct evidence supporting the regulatory relationship between L-2-HG and HIF1A.

Comprehensive analysis of transcriptomic, metabolomic, and proteomic data revealed that L-2-HG and HIF1A play key roles in the brain metastasis of RCC. Compared to previous studies, our research identified 37 differential metabolites associated with the brain metastasis of RCC, among which L-2-HG, related to lactate metabolism, may be closely associated with the occurrence of brain metastasis. Further analysis demonstrated that L-2-HG can enhance the lactylation modification of HIF1A to promote RCC cell resistance to iron death, facilitating cell survival and proliferation in the brain microenvironment, thus promoting brain metastasis (Fig. S7).

The scientific and clinical value of this study lies in its in-depth exploration of the role of L-2-HG in the brain metastasis of RCC and the clarification of its regulatory effect on HIF1A through histone lactylation. These results identified a new mechanistic target for the treatment of RCC brain metastasis. Cell experiments and animal models showed that L-2-HG treatment significantly promoted RCC cell proliferation, migration, and invasion capabilities while suppressing ferroptosis. The brain metastatic microenvironment is typically characterized by hypoxia and metabolic alterations [40, 41], conditions under which L-2-HG may accumulate and further promote metastasis via activation of the HIF1A signaling pathway [27]. Therefore, modulating L-2-HG levels or blocking its effects on HIF1A may represent a novel therapeutic approach for RCC brain metastasis. These findings contribute to a better understanding of the molecular mechanisms underlying RCC brain metastasis and provide important insights for the development of related therapeutic strategies.

The relationship between ferroptosis and tumor metastasis has attracted growing attention. Ferroptosis can act as a tumor-suppressive mechanism, but may also be exploited by tumor cells to facilitate metastasis. Tumor cells often adapt to hypoxic and nutrient-deprived environments through metabolic reprogramming, enhancing survival and promoting dissemination. Ferroptosis disrupts such reprogramming, particularly through suppression of glutathione peroxidase 4, which can increase oxidative stress sensitivity and hinder tumor metastasis [42]. Conversely, cancer cells develop resistance to ferroptosis via multiple mechanisms [43]. For example, upregulation of the antioxidant System Xc^−^ (composed of SLC7A11 and SLC3A2) enhances resistance to ferroptosis [42]. This dual role highlights a complex interplay: induction of ferroptosis may suppress metastasis by promoting cell death and altering the tumor microenvironment while tumor cells may counteract ferroptosis through epithelial-mesenchymal transition and dysregulated iron metabolism to enhance metastatic potential.

Metabolomic analysis revealed significant enrichment of pathways glyoxylate and dicarboxylate metabolism, which are closely associated with the biology of RCC brain metastasis. These pathways are pivotal in cellular energy metabolism and biosynthesis, allowing RCC cells to adapt to the new brain microenvironment during metastasis [44]. Alterations in glyoxylate metabolism may also be linked to mitochondrial function remodeling, such as supporting rapid tumor proliferation by regulating the TCA cycle [45]. These findings suggest that metabolic reprogramming, oxidative stress regulation, and biosynthetic adaptation facilitate RCC brain metastasis.

The present study demonstrated that the L-2-HG–HIF1A axis plays a critical role in RCC brain metastasis, where L-2-HG regulates HIF1A expression via histone lactylation (H3K18la). Previous studies have also shown that L-2-HG promotes a glycolytic phenotype, which supports HIF1A activation [46]. Additionally, L-2-HG stabilizes HIF1A by binding to the catalytic site of prolyl hydroxylase domain-containing protein 2 (PHD2), a key enzyme in HIF1A degradation [47]. This metabolic regulation of HIF1A may be a critical factor in metastatic progression and a potential therapeutic target.

Furthermore, lactate dehydrogenase (LDH), a downstream target gene of HIF1A, is upregulated under hypoxia. Increased LDHA activity promotes lactate production, which serves as a glycolytic byproduct and a substrate donor for histone lactylation [48, 49]. L-2-HG suppresses α-ketoglutarate–dependent dioxygenases such as PHDs, thereby stabilizing HIF1A and further enhancing LDHA expression [50]. Thus, L-2-HG accumulation may affect LDHA activity through multiple mechanisms including metabolic reprogramming, epigenetic regulation, and immunometabolic alterations.

The study found that L-2-HG enhanced H3K18la histone lactylation. Previous studies suggest that L-2-HG may influence intracellular metabolite levels, indirectly affecting lactyl-CoA synthesis and histone lactylation. Under hypoxic conditions, L-2-HG accumulation may promote lactyl-CoA production, thereby increasing histone lactylation [51]. Enzymes such as HBO1 and GTPSCS have been identified as histone lactylation “writers.” HBO1 can directly catalyze histone lactylation [52], while GTPSCS uses nuclear L-lactate to generate lactyl-CoA and forms a complex with p300 to facilitate H3K18la formation [53]. Therefore, L-2-HG-mediated histone lactylation regulation may involve multiple enzymes and epigenetic modifiers. Future studies employing CRISPR or ChIP-seq techniques could help elucidate the regulation of histone lactylation at the HIF1A promoter.

However, this study has certain limitations. Firstly, all experiments were conducted using mouse models, and the direct applicability of these findings to human patients remains to be validated. Secondly, the study explored the effects of L-2-HG on HIF1A and RCC cell proliferation, migration, whether additional regulatory factors influence the role of L-2-HG in RCC brain metastasis remains unclear. Although the L-2-HG–HIF1A axis was confirmed in vivo, changes in key pathway proteins such as HIF1A and H3K18la have not yet been validated in clinical tissue samples, limiting the generalizability of the results. Additionally, the detailed molecular mechanism underlying the regulation of HIF1A by L-2-HG remains unclear and warrants further investigation in future studies.

In terms of future prospects, several directions warrant further investigation. Firstly, further clarification of the regulatory mechanism between L-2-HG and HIF1A is needed, including investigation of the specific impact of lactylation modification on HIF1A function and identification of additional regulatory factors involved in this process. Second, the translational applicability of our findings could be examined using clinical samples and in vitro human RCC brain metastasis models to validate changes in HIF1A and H3K18la expression. Additionally, further exploration of L-2-HG-related metabolic pathways may reveal potential drug targets associated with RCC brain metastasis. Integrating these research aspects is expected to provide more scientific evidence and new treatment strategies for the early diagnosis and treatment of RCC brain metastasis.

## Materials and Methods

### Ethics statement

All animal experiments were conducted in accordance with the regulations and guidelines of our institution’s Animal Ethics Committee and received the required ethical approvals. We made efforts to minimize animal suffering and distress while also minimizing the number of animals required for the experiments. Housing, handling, and experimental procedures adhered to internationally recognized animal welfare standards. Appropriate care was provided throughout the study, and all animals were properly disposed of following experimental procedures.

### Preparation and labeling of tumor cells

To enhance clarity and facilitate comprehension of the experimental workflow involving cellular and animal models, a graphical summary was prepared (Fig. S7), outlining the main experimental procedures, abbreviations, and the number of biological and technical replicates used.

The mouse renal cell carcinoma cell lines, RENCA (CRL-2947, ATCC, USA) and RAG (CCL-142, ATCC, USA), were provided by ATCC. The cells were cultured in RPMI-1640 medium (R4130, Sigma, USA) supplemented with 10% fetal bovine serum (FBS) (F8687, Sigma, USA) and 1% antibiotic-antimycotic solution (HY-K1058, MCE, USA), and maintained in a CO_2_ incubator at 37 °C.

The pGL3-Basic luciferase reporter plasmid (Fig. S1A) was designed and synthesized by Onogen Biological Technology Co., Ltd. When the RENCA cells reached approximately 80% confluency, they were transfected with the luciferase plasmid using Lipofectamine 2000 (11668030, ThermoFisher, USA) (Fig. S1B). After 24 h, Geneticin antibiotic (10131035, ThermoFisher, USA) was added to select stable transfected RENCA-Luc cells. The cells were further cultured, and RENCA-Luc cells in the logarithmic growth phase were seeded into 24-well plates at the following densities: 2 × 10^6^, 1.5 × 10^6^, 1.0 × 10^6^, 0.8 × 10^6^, 0.6 × 10^6^, and 0.4 × 10^6^ cells per well, with 1 mL of culture medium per well. For imaging, 150 μL of 30 mg/mL D-luciferin (L9504, Sigma, USA) was added to each well. After a 5-min incubation, the cells were imaged and analyzed using the IVIS® Spectrum imaging system (PerkinElmer) (Fig. S1C) to confirm cell identification in vitro [54].

### Construction of a mouse model of brain metastasis of RCC

Twenty male BALB/c mice (6–8 weeks old, weighing 16–24 × *g*) and twelve male BALB/c-Nude mice (6–8 weeks old, weighing 19–25 × *g*) were purchased from Changzhou Cavens Experimental Animal Co., Ltd. All mice were housed in SPF-grade animal facilities with a temperature of (23 ± 1) °C, humidity of (55 ± 5%), and a normal light-dark cycle of 12 h. RENCA-Luc cells were resuspended in PBS at a final concentration of 1.25 × 10^7^/mL using PBS.

Brain metastasis models were established using intra-carotid artery injection. Mice were anesthetized with intraperitoneal pentobarbital (40 mg/kg; 1030001, Sigma, USA) and fixed in the supine position on a sterile surgical platform. A support tube was placed beneath the neck to elevate the head, facilitating carotid artery exposure. Fur over the neck was shaved, and the skin was disinfected using iodine solution (109098, Sigma, USA) and 70% ethanol. Under a surgical microscope, a midline vertical neck incision was made, and subcutaneous tissue and fat were bluntly dissected to expose the left carotid sheath. After opening the left carotid artery sheath, the left common carotid artery (CCA), left internal jugular vein, and left vagus nerve were carefully separated. The internal and external carotid arteries (ICA and ECA) were further exposed by blunt dissection at the level of the thyroid cartilage (Fig. S8A, B). The CCA was ligated close to the heart end, and the ECA was ligated. A loose ligature was placed distally to the injection site in the CCA, and a PBS-soaked cotton pad was positioned underneath the CCA to stabilize the vessel. Another ligature was prepared proximally for a loop tie. The CCA was lifted, and a 33-gauge needle was inserted diagonally into the carotid artery lumen under the microscope. Upon insertion, fresh red blood was observed, and the injection of tumor single-cell suspension was performed by applying pressure with a gauze piece (Fig. S8C). In the RCC group, 50 μL of tumor cell suspension was slowly injected into the CCA over more than 2 min, while the Sham group received an equal amount of PBS as a control. The successful injection was indicated by a change in the color of the front-end blood vessel. Before needle withdrawal, the distal loop ligature was tightened to prevent reflux. Excess sutures were trimmed, and the muscle, fat, and skin layers were closed sequentially. The incision site was disinfected, and mice were placed in a heated cage for recovery. Upon regaining full mobility, mice received a subcutaneous injection of ibuprofen (I4883, Sigma, USA) as a postoperative analgesic and were maintained under standard conditions. Health status was closely monitored. Mice were euthanized based on behaviors such as rapid weight loss, hunching, inability to eat or drink, severe bilateral or unilateral limb coordination impairment, and inability to move. Survival time was calculated, and brain tissue was harvested for subsequent experiments at the end of the experiment [21].

### In vivo fluorescence imaging

Beginning on the third day after model establishment, in vivo fluorescence imaging was performed weekly using the IVIS® Spectrum imaging system to monitor tumor growth and metastasis. Prior to imaging, mice were anesthetized by intraperitoneal injection of D-luciferin at a dose of 80 mg/kg. Ten minutes after injection, mice were placed in the imaging chamber for optical scanning. Bioluminescent signals were captured, and signal intensity was recorded. Image brightness and distribution were analyzed using software to evaluate the distribution, growth, and metastasis of tumor cells in the brain [55].

### H&E staining

At the end of the experiment, the mice were euthanized. The brain tissues were carefully removed, washed with PBS, and fixed with 4% paraformaldehyde (P6148, Sigma, USA). Fixed tissues underwent standard histological processing, including dehydration, clearing, and embedding and were sectioned at a thickness of 5 μm. The tissue sections were mounted on glass slides and dried at 45 °C. After deparaffinization with xylene (247642, Sigma, USA) and graded alcohol washes, sections were rinsed with distilled water for 2 min. Hematoxylin staining (H3136, Sigma, USA) was performed for 5 min, followed by differentiation in 1% hydrochloric acid ethanol for 3 s and eosin staining (5%, 861006, Sigma, USA) for approximately 2 min. Dehydration and clearing were completed, and sections were mounted using neutral resin. Histological features were examined using an optical microscope to evaluate the metastasis of RCC cells in the brain [56].

### Immunohistochemical staining (IHC)

The tissue section preparation method was performed as previously described. Tissue sections were boiled in a solution containing 0.01 M citrate buffer (P4809, Sigma, USA) for 15–20 min for antigen retrieval. Following that, the sections were incubated in 3% H_2_O_2_ at room temperature for 30 min to inactivate endogenous peroxidase. Sections were then incubated with goat serum blocking solution (S26-M, Sigma, USA) for 20 min. Excess serum was removed, and primary antibodies (Table S2) were added and incubated at room temperature for 1 h. After PBS washing, the sections were incubated with a secondary antibody against IgG (ab6721, 1:1000, Abcam, UK) at 37 °C for 20 min, followed by another PBS wash. After that, the sections were treated with SP (streptavidin-peroxidase) and incubated at 37 °C for 30 min, followed by PBS wash. Subsequently, DAB (P0202, Beyotime Biotechnology Co., Ltd) was added for color development, and the sections were stained for 5–10 min. After a 10-min water wash to stop the reaction, the sections were stained for another 2 min and differentiated using hydrochloric alcohol. Following a 10-min wash, gradient alcohol dehydration (xylene as the clearing agent) was performed, and then 2–3 drops of neutral resin were added for slide mounting. The sections were observed under an upright microscope, and the protein expression positive regions within the regions of interest were measured using an image analysis system (Aperio Scanscope System, Vista, CA). The average optical density values were calculated [56].

### High-throughput metabolomics sequencing and data analysis

Tumor tissues were isolated from RCC mice and Sham group mice, with 4 mice in each group. The tissues were frozen in liquid nitrogen and stored at −80 °C. Prior to sample processing, the tissues were quickly ground into powder under liquid nitrogen. For every 1 mg of brain tissue, 5 µL of 0.1% formic acid (F0507, Sigma, USA) was added, homogenized for 30 s, centrifuged at 14,000 rpm for 10 min, and the supernatant was collected. The supernatant was then mixed with 4 times its volume of acetonitrile (34851, Sigma, USA), vortexed for 30 s, centrifuged at 14,000 rpm for 10 min, and the supernatant was collected and stored at −20 °C.

An LC20 ultra-high-performance liquid chromatography system (Japan Shimadzu) and Triple TOF-6600 mass spectrometer (AB Sciex) were used for plasma metabolomics analysis. A Waters ACQUITY UPLC HSS T3 C18 (100 × 2.1 mm, 1.8 μm) column was used for chromatographic analysis. The column temperature was maintained at 40 °C, and the mobile phase was delivered at a flow rate of 0.4 mL/min. The mobile phase consisted of an acetonitrile-water solution containing 0.1% formic acid. The gradient elution program for mobile phase B was as follows: 5%, 0.0–11.0 min; 90%, 11.1–12.0 min; 5%, 12.1–14 min. The eluate was directly introduced into the mass spectrometer without splitting [57].

The mass spectrometry conditions were as follows: ionization voltage, 5500 V; capillary temperature, 550 °C; spray gas flow rate, 50 psi; auxiliary heating gas flow rate, 60 psi. OPLS-DA and permutation tests (100 permutations) were used to analyze the preprocessed data to prevent overfitting. Metabolites with VIP scores greater than 1 in the OPLS-DA model were identified as differentially expressed metabolites. Moreover, combining univariate analysis, metabolites with fold changes greater than or equal to 2 and a *P*-value less than 0.05 in Student’s *t*-test were selected as the final differential metabolites. The related metabolic pathways were identified using MetaboAnalyst (version 5.0) [58].

### Transcriptome sequencing and data analysis

Brain tissues from three mice per group (Sham and RCC) were collected for transcriptomic analysis. Total RNA was isolated using a Trizol reagent (15596026, Invitrogen, California, USA). The MGIEasy rRNA Removal Kit (1000005953, MGI, China) was employed to remove ribosomal RNA (rRNA) from the total RNA, and the resulting RNA was fragmented into approximately 300 bp fragments using ion fragmentation. First-strand cDNA was synthesized from the fragmented RNA using a 6-base random primer and reverse transcriptase. Subsequently, second-strand cDNA was synthesized using the first-strand cDNA as a template. After library construction, PCR amplification was performed to enrich the library fragments, with 450 bp fragments selected as the library. The quality of the library was assessed using the Agilent 2100 Bioanalyzer, and the total concentration and effective concentration of the library were determined. Based on the effective concentration and the required sequencing depth, libraries with different index sequences were proportionally pooled. The pooled libraries were denatured under alkaline conditions to generate single-stranded DNA for sequencing.

After RNA extraction, purification, and library construction, paired-end sequencing (PE sequencing) was performed using the Illumina HiSeq platform. Adapter sequences were removed from raw reads using Cutadapt (v1.18), and low-quality sequences were filtered out using fqtools (v0.1.6), resulting in high-quality data (Clean Reads). All subsequent analyses were based on the Clean Reads data. The Q30 quality value of the Clean Reads data was calculated. The filtered Clean Reads sequences were aligned to the mouse genome (Mus musculus GRCm38.90) using the HiSAT2 software (v.2.1.0).

Transcript annotation was performed using custom Perl scripts. Differential gene expression analysis between the RCC and Sham groups was conducted using the R package “DESeq,” with significance thresholds set at |logFC| > 1 and *P* < 0.05. Differential analysis heatmaps, volcano plots, and Venn diagrams were generated using the OmicShare online analysis tool (https://www.omicshare.com). Lastly, gene retrieval related to “Renal cell carcinoma” and “Brain metastases” was retrieved from the GeneCards database (https://www.genecards.org) and ranked by relevance score. The top 500 genes were selected as disease-related genes [59].

### Bioinformatic analysis of the TCGA database

Gene expression profiles and clinical data of RCC patients were retrieved from the Cancer Genome Atlas (TCGA) database (https://www.cancer.gov). Expression data were downloaded in FPKM format. Perl scripts were utilized to annotate and correct the gene expression data. Subsequently, the “edgeR” package in the R software was employed for the differential analysis of paired samples to validate the differential expression of identified key genes associated with RCC brain metastasis. Moreover, the R software packages “survival” and “timeROC” were used to conduct survival analysis and ROC curve plotting to investigate the correlation between these key genes and patient prognosis [60].

### Sample preparation and determination for proteomics

Brain tissues from three mice per group (Sham and RCC) were ground into powder in liquid nitrogen and transferred to 5 mL centrifuge tubes. A phenol extraction buffer containing 10 mM DTT (R0861, Solarbio, Beijing, China), 1% protease inhibitor cocktail (P6731, Solarbio, Beijing, China), and 2 mM EDTA (E1170, Solarbio, Beijing, China) was added, followed by ultrasonication using a SCIENTZ-IID ultrasonic cell disruptor (SCIENTZ, Ningbo, China) in an ice bath for eight cycles. Next, an equal volume of pH 8.0 Tris-saturated phenol (HC1380, Vander Biological, Beijing, China) was added and mixed for 4 min using a vortex mixer. The tubes were then centrifuged at 4 °C and 5000 × *g* for 10 min, and the upper phenol layer was transferred to new centrifuge tubes. To the phenol solution, an equal volume of 0.1 M ammonium sulfate saturated methanol (101217, Merck, USA) was added with a volume ratio of 1:5 and left overnight to precipitate proteins. The supernatant was discarded after centrifugation at 4 °C for 10 min. Lastly, the protein precipitate was washed once with cold methanol and three times with cold acetone. The washed protein was dissolved in 8 M urea (U8020, Solarbio, Beijing, China), and the protein concentration was determined using the BCA assay kit (P0012, Beyotime, Shanghai, China) following the manufacturer’s instructions [61].

### Enzymatic digestion and mass analysis

For each sample, 50 µg of protein was subjected to enzymatic digestion. The protein solution was mixed with DTT to achieve a concentration of 5 mM and incubated at 56 °C for 30 min. Acetonitrile was then added to a concentration of 11 mM and incubated at room temperature for 15 min. The urea concentration was subsequently diluted to below 2 M, and trypsin (25200056, Thermo Fisher Scientific, USA) was added at a mass ratio of 1:50 (w/w), followed by overnight digestion at 37 °C. After trypsin digestion, additional trypsin was added at a mass ratio of 1:100 (trypsin: protein), and the digestion was continued for 4 h.

Following trypsin digestion, the peptides were desalted using a HyperSep™ C18 purification column (60108-302, Thermo Fisher Scientific, USA) and then dried under vacuum. The peptides were reconstituted in 0.5 M TEAB (90114, Thermo Fisher Scientific, USA) and labeled using the TMT reagent kit (90064CH, Thermo Fisher Scientific, USA). In simple terms, a unit of TMT reagent was thawed and reconstituted in acetonitrile (113212, Merck, USA). The peptide mixture was then incubated at room temperature for 2 h and desalted and dried using a vacuum centrifuge. Equal amounts of labeled peptides from each group were combined, and the dried peptides were reconstituted using Pierce™ high-pH reversed-phase peptide separation kit (84868, Thermo Fisher Scientific, USA). Finally, the samples were collected and merged into 15 fractions, and the dried peptides from each fraction were reconstituted using 0.1% formic acid (159002, Sigma, USA).

For each sample, 2 µg of peptides were separated using the nano-UPLC liquid chromatography system Easy nLC 1200 (Thermo Fisher Scientific, USA). Peptides were first loaded onto a trap column (C18, 100 μm × 20 mm, 5 μm) and then separated on an analytical column (C18, 75 μm × 150 mm, 3 μm) at a flow rate of 300 nL/min. The mobile phase A consisted of a 0.1% formic acid aqueous solution, while the mobile phase B was a 0.1% formic acid water-acetonitrile solution (containing 95% acetonitrile). The gradient elution program was as follows: 0 → 2 min, 2% → 8% B; 2 → 71 min, 8% → 28% B; 71 → 79 min, 28% → 40% B; 79 → 81 min, 40% → 100% B; 81 → 90 min, 100% B. The separated peptides were analyzed by Q-Exactive HFX mass spectrometer (Thermo Fisher Scientific, USA). The analysis duration was set to 60 min, with an electrospray voltage of 2.1 kV, positive ion mode, a mass range for precursor ion scans from 350 to 1200 m/z, a first-level mass resolution of 60000@m/z 200, an AGC target of 3e6, and a first-level maximum IT of 30 ms. The second-level mass resolution was set to 15000@m/z 200, with an AGC target of 1e6, a second-level maximum IT of 25 ms, MS2 Activation Type: HCD, an isolation window of 20 Th, and a normalized collision energy of 32 [62].

### Proteomic database retrieval and data processing

LC-MS/MS data obtained were processed using MaxQuant software (v.1.5.2.8), including peptide identification and protein quantification. The UniProt 14.1 (2009) Protein sequences were matched against the UniProt 14.1 (2009) *Gossypium hirsutum* database combined with a reverse decoy database for tandem mass spectrometry searches. Trypsin/P was designated as the cleaving enzyme, with a maximum of two missed cleavage sites allowed. The initial search was performed with a mass tolerance of 20 ppm, followed by a subsequent search with a primary mass tolerance of 5 ppm and fragment ion mass tolerance of 0.02 Da. A peptide spectrum match distribution, along with a peptide false discovery rate (FDR) ≤ 0.01, protein FDR ≤ 0.01, and peptide score distribution, was used as filtering criteria. Differential expression proteins were selected using the “Limma” package in R software with a threshold of *P*-value < 0.05.

Functional Enrichment Analysis: GO and KEGG pathway enrichment analysis was conducted using the “ClusterProfiler” package in R software to identify significantly enriched biological processes, molecular functions, and cellular components among the differentially expressed proteins.

PPI Protein-Protein Interaction Network Analysis: A PPI network related to differential proteins was constructed using the STRING database. Subsequently, Cytoscape software was employed for network visualization, modularization, and subnetwork analysis [63, 64].

### Cell culture and grouping

RCC cell lines were cultured under previously established conditions. Firstly, the cell suspension was gently pipetted to ensure even distribution, followed by cell counting. The cell number was adjusted to approximately 1.5 × 10^5^ cells per well and seeded into six-well plates. Subsequently, the six-well plate was gently agitated to achieve uniform distribution. Upon reaching 50–60% confluence, cells were grouped and subjected to respective treatments. For the L-2-HG group, 2 mL of L-2-HG with a concentration of 5 mM (CY18816, ChemeGen, USA) was added to each well [65, 66]. For the L-2-HG + Oxa group, 2 mL of a mixture including 5 mM L-2-HG and 5 mM oxaloacetate sodium (O2751, Sigma, USA) was added to each well. All drug solutions were prepared in complete culture medium and filtered through a 0.22 μm membrane. Three biological replicates were established for each group. After 24 h of continuous culture, the cells were collected for subsequent experiments [67].

### Western blot

Total protein from cells and tissues was extracted using a protein extraction kit (Bestbio, BB3101, China). Protein concentration was determined using the BCA assay kit. Subsequently, the proteins were separated using SDS-PAGE electrophoresis and transferred onto a PVDF membrane (IPVH85R, Millipore, Darmstadt, Germany). At room temperature, the membrane was blocked with 5% BSA for 1 h. Then, immunoblotting was performed using the corresponding primary and secondary antibodies. The membrane was washed with TBST for 5 min and repeated three times. Chemiluminescent detection was performed using an imaging system. Protein quantification analysis was conducted using ImageJ 1.48 u software (V1.48, National Institutes of Health, USA). Protein quantification was determined by comparing the grayscale values of the proteins to the grayscale ratio of histone H3 or β-actin. Details of antibody sources are listed in Table S3 [68, 69].

### Immunofluorescence staining

RCC cells were seeded in a 24-well plate at a density of 5 × 10^4^ cells per well and incubated 37 °C, 5% CO_2_ for 24 h. After removing the culture medium, the cells were washed three times with PBS. Then, 4% formaldehyde (47673, Sigma, USA) was added to each well at room temperature for 20 min, followed by three additional PBS washes. Subsequently, the cells were treated with 0.1% Triton ×-100 (×100, Sigma, USA) for 10 min, followed by another three washes with PBS. The cells were incubated with PBS containing 5% BSA (V900933, Sigma, USA) at room temperature for 1 h to block nonspecific binding. Rabbit anti-H3K18la antibody (PTM-1406RM, 1:100, PTM BIO, China) diluted in PBS was added, and the cells were incubated overnight at 4 °C. After removing the primary antibody, cells were washed three times with PBS for 5 min each. A fluorescently labeled secondary antibody (ab150077, 1:200, Abcam, UK) was added, and the cells were incubated in the dark at room temperature for 1–2 h. The secondary antibody was removed, and the cells were washed again three times with PBS for 5 min each. DAPI stain (D1306, ThermoFisher, USA) was added for nuclear staining and incubated for 10 min. After removing DAPI, the cells were washed three times. Finally, the cells were sealed with an anti-fading mounting medium. Images were captured using a laser scanning confocal microscope (Zeiss LSM 880). Image analysis and quantification of the fluorescence signal intensity of the cells were performed using either the microscope’s built-in software or a third-party software such as ImageJ [68, 69].

### Chromatin immunoprecipitation (ChIP)

Treated cells were fixed with 1% formaldehyde at room temperature for 15 min to induce cross-linking. Cell lysis was performed using RIPA lysis buffer containing protease and nuclease inhibitors to release chromatin. Ultrasonic disruption was carried out using a sonicator to break the chromatin. PEG precipitation was then performed to precipitate chromatin with PEG8000 (P8260, Solarbio, China) and transferred to a new tube. Chromatin immunoprecipitation was performed by adding rabbit anti-H3K18la antibody (PTM-1406RM, 1:50, PTM BIO, China) or rabbit anti-mouse IgG (ab6709, 1:100, Abcam, UK) to the chromatin mixture to capture the specific transcription factor binding. Magnetic bead precipitation was performed to capture the target antibody-chromatin complex using protein A/G magnetic beads (P2012, Beyotime, China). A sequential wash with buffers of varying salt concentrations was applied to eliminate nonspecific interactions and impurities. Crosslinking was reversed by heating at 65 °C for 0.5 h in a buffer containing 5 M NaCl, 20 mg/ml proteinase K (1.24568, Sigma, USA), and 10% SDS (L3771, Sigma, USA). DNA purification was performed using the QIAGEN PCA Purification Kit (28106, QIAGEN, Germany) for subsequent qPCR detection, as described in the RT-qPCR section [70]. IgG was used as a negative control, and YTHDF2 served as a positive control for the H3K18la antibody [35]. The ChIP-qPCR primers for the HIF1A promoter were designed to amplify the −830 to −123 region.

### RT-qPCR

Total RNA was extracted following standard protocols, and the concentration and purity were assessed using a NanoDrop 2000 spectrophotometer (ND-2000, ThermoFisher, USA). Reverse transcription of mRNA into cDNA was performed using the PrimeScript RT Reagent Kit (RR047A, Takara, Japan). qPCR was conducted using SYBR Green Master Mix (RR420A, Takara, Japan) or equivalent reagents. Fluorescence signals were measured in a real-time PCR system with three technical replicates per sample. β-actin served as the internal control. Relative gene expression was calculated using the 2^−ΔΔCt^ method. The formula was as follows: ΔΔCT = ΔCt experimental group—ΔCt control group, where ΔCt = Ct target gene—Ct reference gene. Ct refers to the cycle number at which fluorescence exceeded the threshold and amplification entered the exponential phase [71]. Primers were designed using the Primer-BLAST tool, and sequences are listed in Table S4.

### HIF1A cell transfection and grouping

Lentiviral vectors encoding RFP-labeled sh-HIF1A-1 and sh-HIF1A-2 were designed and synthesized by Suzhou Genesil Biotechnology Co., Ltd., each with a viral titer of 5 × 10^8^ TU. The corresponding control group, sh-NC, was transfected with an empty lentiviral vector with a virus titer of 5 × 10^8^ TU (Table S5). Briefly, mouse RCC cells, RENCA and RAG, were seeded in a 96-well plate. Upon reaching 50% confluence, cells were infected with different groups of lentiviruses. Additionally, 5 μg/ml of Polybrene (TR1003, Sigma, USA) was added. After 24 h, the culture medium was replaced. Transfection efficiency was evaluated based on RFP fluorescence observed at 3–4 days post-infection. Cells were then used for subsequent experiments.

Based on the experimental purpose, specific treatments were applied to the transfected cells. The cells transfected with sh-NC, sh-HIF1A-1, and sh-HIF1A-2 were treated with the corresponding lentiviral vectors. sh-HIF1A-1, which showed superior silencing efficiency, was selected for follow-up experiments. L-2-HG+sh-NC and L-2-HG+sh-HIF1A groups were treated with L-2-HG after transfection with sh-NC or sh-HIF1A lentiviruses. L-2-HG+sh-HIF1A + DFO group was treated with both L-2-HG and DFO (D9533, Sigma, USA) simultaneously, with a final concentration of 100 μM [72].

### Cell proliferation assay using CCK-8

For cell proliferation analysis, cells in the healthy growth state were seeded in a 96-well plate at a density of 8 × 10^3^ cells per well and incubated in a CO_2_ incubator. At 0, 24, 48, and 72 h, 10 μL of CCK-8 solution (96992, Sigma, USA) was added to each well. After incubating for 1 h at 37 °C in a humidified incubator, the absorbance of each sample was measured at 450 nm using an Epoch microplate spectrophotometer (Bio-Tek, Winooski, VT, USA) [73]. Each group had 6 replicates, and the experiment was repeated three times.

### Transwell experiment

The Transwell invasion experiment was conducted after cells had been treated for 48 h. A 50 μL layer of matrix gel (354234, BD Biosciences, USA) was added to the upper chamber and incubated at 37 °C for 30 min to allow solidification. The coated chamber was then washed with a medium devoid of FBS, and cells were diluted to a concentration of 2.5 × 10^4^ cells per mL. Subsequently, 100 μL of cell suspension was added to each well in the upper chamber, while 500 μL of medium containing 10% FBS was added to the lower chamber. After 24 h, the chamber was removed, and cells in the upper chamber were gently removed using a cotton swab. Fixation was then performed using 4% PFA at room temperature for 30 min, followed by staining with 0.1% crystal violet for 30 min. Five random fields per membrane were imaged and counted under an inverted microscope (IXplore Pro, Olympus, Japan). The experiment was repeated three times [73].

### Scratch assay

Cells were seeded into 24-well plates and cultured until confluence. A sterile 200 μL pipette tip was used to create linear scratches, followed by PBS washing to remove detached cells. Serum-free RPMI 1640 medium was added, and the cells were incubated for an additional 24 h. Images were captured at 0 and 24 h using a 5× objective under an inverted microscope. Scratch areas were analyzed using Image J software, and the percentage of wound closure was calculated [73].

### Measurement of cellular iron death levels

The levels of Fe^2+^ in cells or tissues were determined using the Iron Assay Kit (ab83366, Abcam, UK). Samples were washed with cold PBS, homogenized, and lysed in an iron assay buffer. The supernatant was collected after centrifugation at 16,000 × *g* for 10 min. An iron-reducing reagent was added to both the samples and standard solutions, followed by incubation at 37 °C for 30 min. Subsequently, the iron probe was added, and the mixture was further incubated in the dark at 37 °C for 60 min. Absorbance was measured at 593 nm using an ELISA microplate reader.

ROS levels were evaluated using the DCFDA Cellular ROS Detection Assay Kit (ab113851, Abcam, UK). The cells or tissues plated in a black 96-well plate were homogenized, and the supernatant was collected. DCFDA labeling was added, and the mixture was incubated at 37 °C for 30 min. The fluorescence intensity of DCF was measured using a fluorescence microplate reader (BioTek) at an excitation wavelength of 485 nm and an emission wavelength of 535 nm.

For the supernatant of homogenized mouse tumor tissues or the tested tumor cells, GSH (A006-2-1, Nanjing Jiancheng) and MDA (A003-1-2, Nanjing Jiancheng) were measured separately according to the instructions of the assay kit [74].

### Construction of lung metastasis model

A lung metastasis model was established using fluorescence-labeled RCC cells (RENCA-Luc) and BALB/c-Nude male nude mice. The fluorescence-labeling method was described previously. 1 × 10^7^ RENCA-Luc cells were injected into the mice via the tail vein. The mice were divided into the NC group and L-2-HG group, with 10 mice in each group. The L-2-HG group received daily intraperitoneal injections of 10 mg/kg L-2-HG, while the NC group received an equal volume of PBS.

In vivo fluorescence imaging was conducted weekly, as previously described. At the end of the third week after injection, mice were euthanized, and tumor tissues were collected for counting lung metastases. The levels of HIF1A, H3K18la, and Ki67 were detected by immunohistochemistry staining, as previously described [75].

### Statistical analysis

Descriptive statistics, including mean, median, standard deviation, and range, were initially calculated. Comparisons between the two groups were performed using independent-sample *t*-tests. One-way analysis of variance was used for comparisons involving three or more groups, while multifactorial comparisons were analyzed using multivariate analysis of variance. Kaplan–Meier survival curves were employed for survival time data, and the log-rank test was used to compare differences in survival curves. Assessment of the relationship between continuous variables was conducted using the Pearson correlation coefficient. Linear or logistic regression analyses were performed for predictive modeling. When data failed to meet normality or homogeneity of variance assumptions, non-parametric tests such as the Mann–Whitney *U* test or Kruskal–Wallis test were applied. All statistical analyses were performed using SPSS (IBM Corporation) or R software. A *p*-value less than 0.05 was considered statistically significant. All figures were generated using GraphPad Prism software (GraphPad Software, Inc.).

## Supplementary information

Supplementary Tables and Figures

Full and uncropped western blots

## Data Availability

The experimental data sets generated and/or analyzed during the current study are available from the corresponding author upon reasonable request.
