# Supplementary material for: Novel role of L-2-HG in regulating HIF1A signaling pathway and iron death resistance in renal cancer brain metastasis
Source: Cell Death Dis. 2025 Nov 6;16(1):798. doi: 10.1038/s41419-025-08068-z (PMC12592421; doi:10.1038/s41419-025-08068-z)
Supplement: Supplementary file 1 — Supplementary Tables and Figures [file 41419_2025_8068_MOESM1_ESM.docx]

**Table S1. Information of differential metabolites**

| **Metabolites** | **Formula** | **Structure** | **m/z** |
| --- | --- | --- | --- |
| 13S-HODE | C_18_H_32_O_3_ | 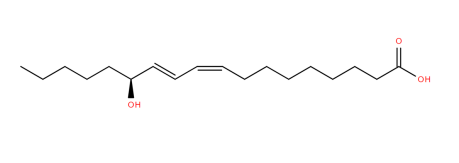 | 296.2346 |
| 24,25-dihydrolanosterol | C_30_H_52_O | 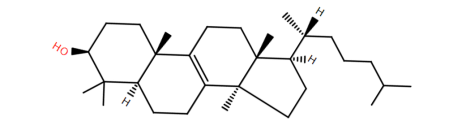 | 428.4013 |
| 4-Hydroxyphenyllactic acid | C_9_H_10_O | 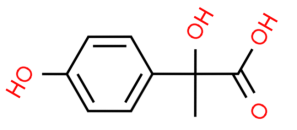 | 182.0579 |
| AICAR | C_9_H_15_N_4_O_8_P | 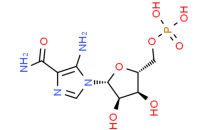 | 338.0627 |
| Aminoisobutyric acid | C_4_H_9_NO_2_ | 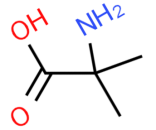 | 103.0633 |
| CAR 10:0 | C_17_H_33_NO_4_ | 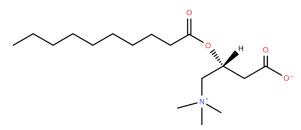 | 315.2404 |
| CAR 12:0 | C_19_H_37_NO_4_ | 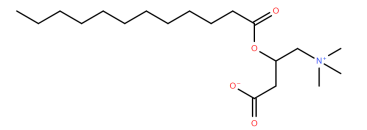 | 343.2717 |
| CAR 12:1 | C_19_H_35_NO_4_ | 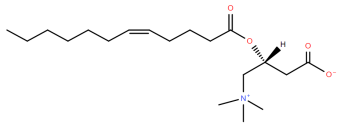 | 341.2561 |
| CAR 14:0 | C_21_H_41_NO_4_ | 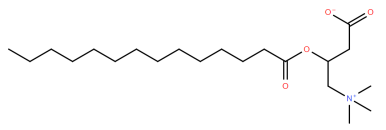 | 371.3030 |
| CAR 14:0_1 | C_21_H_41_NO_4_ | 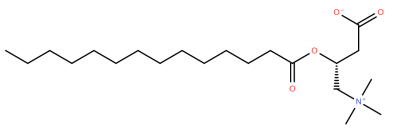 | 371.3030 |
| CAR 14:1 | C_21_H_39_NO_4_ | 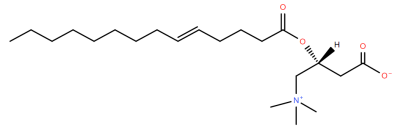 | 369.2874 |
| CAR 16:0 | C_23_H_45_NO_4_ | 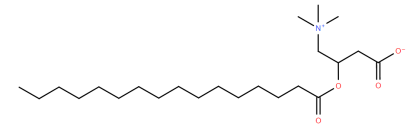 | 399.3343 |
| CAR 16:0;O | C_23_H_45_NO_5_ | 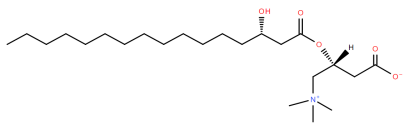 | 415.3292 |
| CAR 16:1 | C_23_H_43_NO_4_ | 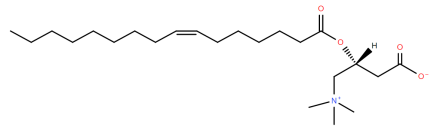 | 397.3187 |
| CAR 18:0 | C_25_H_49_NO_4_ | 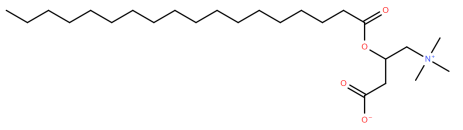 | 427.3656 |
| CAR 18:1 | C_25_H_47_NO_4_ | 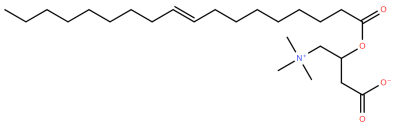 | 425.3500 |
| CAR 18:1;O2 | C_25_H_47_NO_6_ | 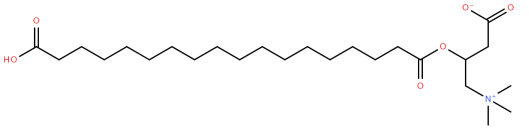 | 457.3398 |
| CAR 18:2 | C_25_H_45_NO_4_ | 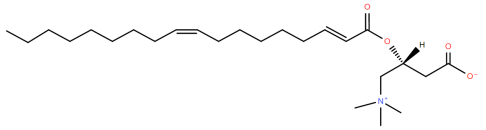 | 423.3343 |
| CAR 8:0 | C_15_H_29_NO_4_ | 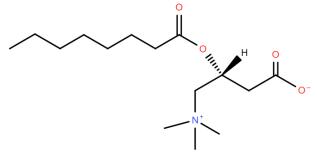 | 287.2091 |
| Carbamoylphosphate | CH_2_NO_5_P | 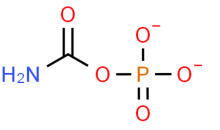 | 138.9682 |
| Cystine | C_6_H_12_N_2_O_4_S_2_ | 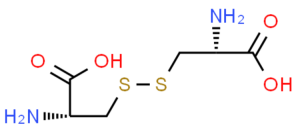 | 240.0238 |
| Cytidine | C_9_H_13_N_3_O_5_ | 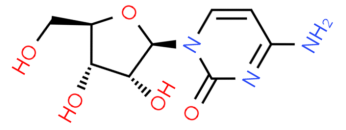 | 243.0855 |
| Dextroamphetamine | C_9_H_13_N | 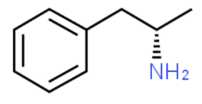 | 135.1048 |
| Docosahexaenoic acid | C_22_H_32_O_2_ | 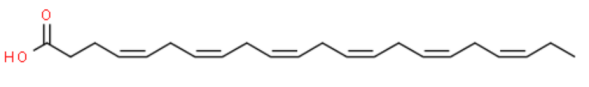 | 328.2402 |
| Dopamine | C_8_H_11_NO_2_ | 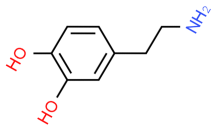 | 153.0790 |
| Gamma-Aminobutyric acid | C_4_H_9_NO_2_ | 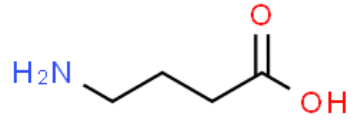 | 103.0633 |
| Glycine | C_2_H_5_NO_2_ | 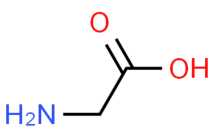 | 75.0320 |
| GlcCer(d18:1/20:0) | C_44_H_85_NO_8_ | 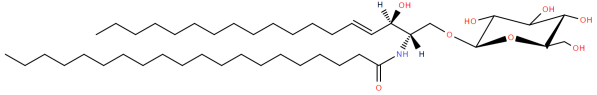 | 755.6270 |
| GlcCer(d18:1/24:1(15Z)) | C_48_H_91_NO_8_ | 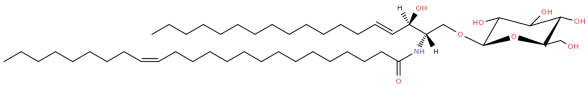 | 809.6739 |
| Hypoxanthine | C_5_H_2_N_4_O | 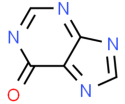 | 134.0229 |
| L-2-Hydroxyglutaric acid | C_5_H_8_O_5_ | 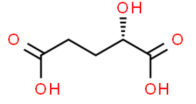 | 148.0372 |
| PC 36:4 | C_44_H_80_NO_8_P | 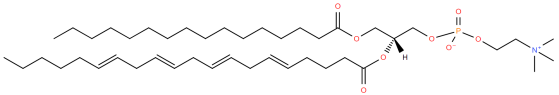 | 781.5616 |
| PC O-36:5 | C_44_H_80_NO_7_P | 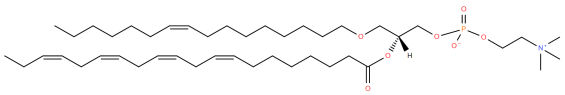 | 765.5667 |
| PC O-36:6 | C_44_H_78_NO_7_P | 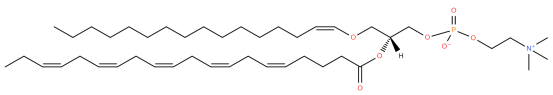 | 763.5510 |
| Serine | C_3_H_7_NO_3_ | 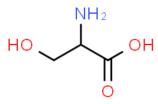 | 105.0426 |
| SM(d18:1/25:0) | C_48_H_97_N_2_O_6_P | 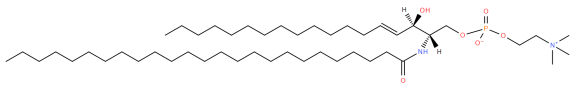 | 828.7079 |
| sn-Glycero-3-phosphocholine | C_8_H_20_NO_6_P | 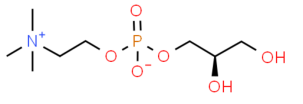 | 257.1028 |

**Table S2. Antibody information in IHC**

| **Antibody** | **Product No.** | **Dilution Ratio** | **Manufacturer** |
| --- | --- | --- | --- |
| Rabbit polyclonal to HIF-1 alpha | ab114977 | 1:100 | Abcam |
| Anti-L-Lactyl-Histone H3 (Lys18) Rabbit mAb | PTM-1406RM | 1:200 | PTM BIO |
| Rabbit polyclonal to Ki67(IHC) | ab15580 | 1:100 | Abcam |

**Table S3. Antibody information in Western Blot**

| **Antibody** | **Product No.** | **Dilution Ratio** | **Manufacturer** |
| --- | --- | --- | --- |
| Anti-L-Lactyl Lysine Rabbit mAb | PTM-1401RM | 1:1000 | PTM BIO |
| Anti-L-Lactyl-Histone H3 (Lys18) Rabbit mAb | PTM-1406RM | 1:1000 | PTM BIO |
| Rabbit monoclonal to Histone H3 | ab176842 | 1:1000 | Abcam |
| Rabbit monoclonal to HIF-1 alpha | ab179483 | 1:1000 | Abcam |
| Rabbit monoclonal to beta Actin | ab213262 | 1:1000 | Abcam |
| Goat Anti-Rabbit IgG | ab6721 | 1:2000 | Abcam |

**Table S4. Primer sequence**

| **Gene** | **Primer Sequence (5’-3’)** |
| --- | --- |
| HIF1A(mouse) | Forward: CTTGACAAGCTAGCCGGAGG |
|  | Reverse: AAGAGACAAGTCCAGAGGCG |
| HIF1A(ChIP) | Forword: GCATCCATGTGTGGAGACCA |
|  | Reverse: CTGCAAACTCCAAGGCAACC |
| β-actin(mouse) | Forword: CACTGTCGAGTCGCGTCC |
|  | Reverse: CGCAGCGATATCGTCATCCA |

**Table S5. shRNA sequence**

| **shRNA** | **Sequence (5’-3’)** |
| --- | --- |
| sh-NC | GCCAATATACAGATTCAAGAT |
| sh-HIF1A-1 | GCCACTTTGAATCAAAGAAAT |
| sh-HIF1A-2 | GCCGCTCAATTTATGAATATT |


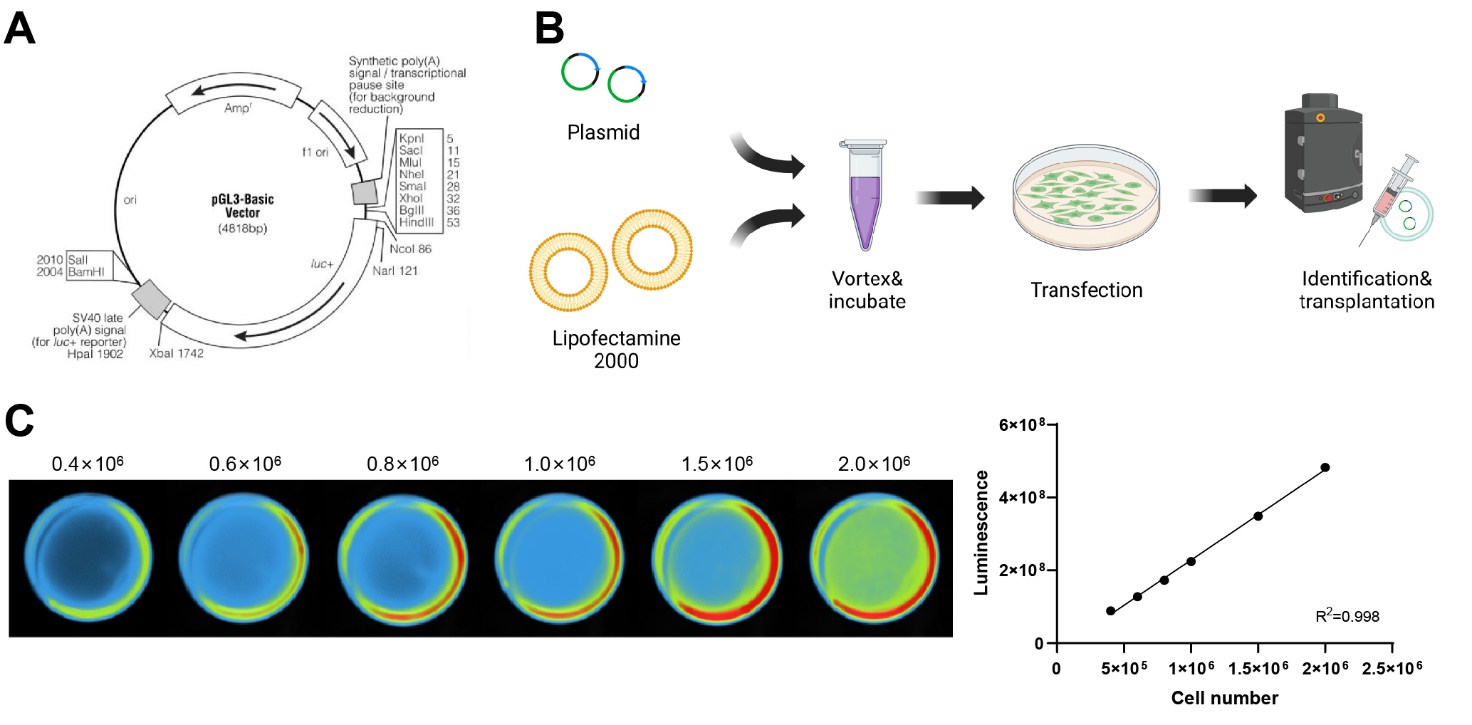


**Figure S1. Preparation and identification of fluorescently labeled RCC cells in mice.**

Note: (A) Schematic diagram of the pGL3-Basic plasmid structure (Created with BioRender.com); (B) Transfection procedure of the luciferase reporter plasmid (Created with BioRender.com); (C) *In vitro* cell imaging of transfected cells with different cell numbers and correlation analysis between fluorescence intensity and cell number.


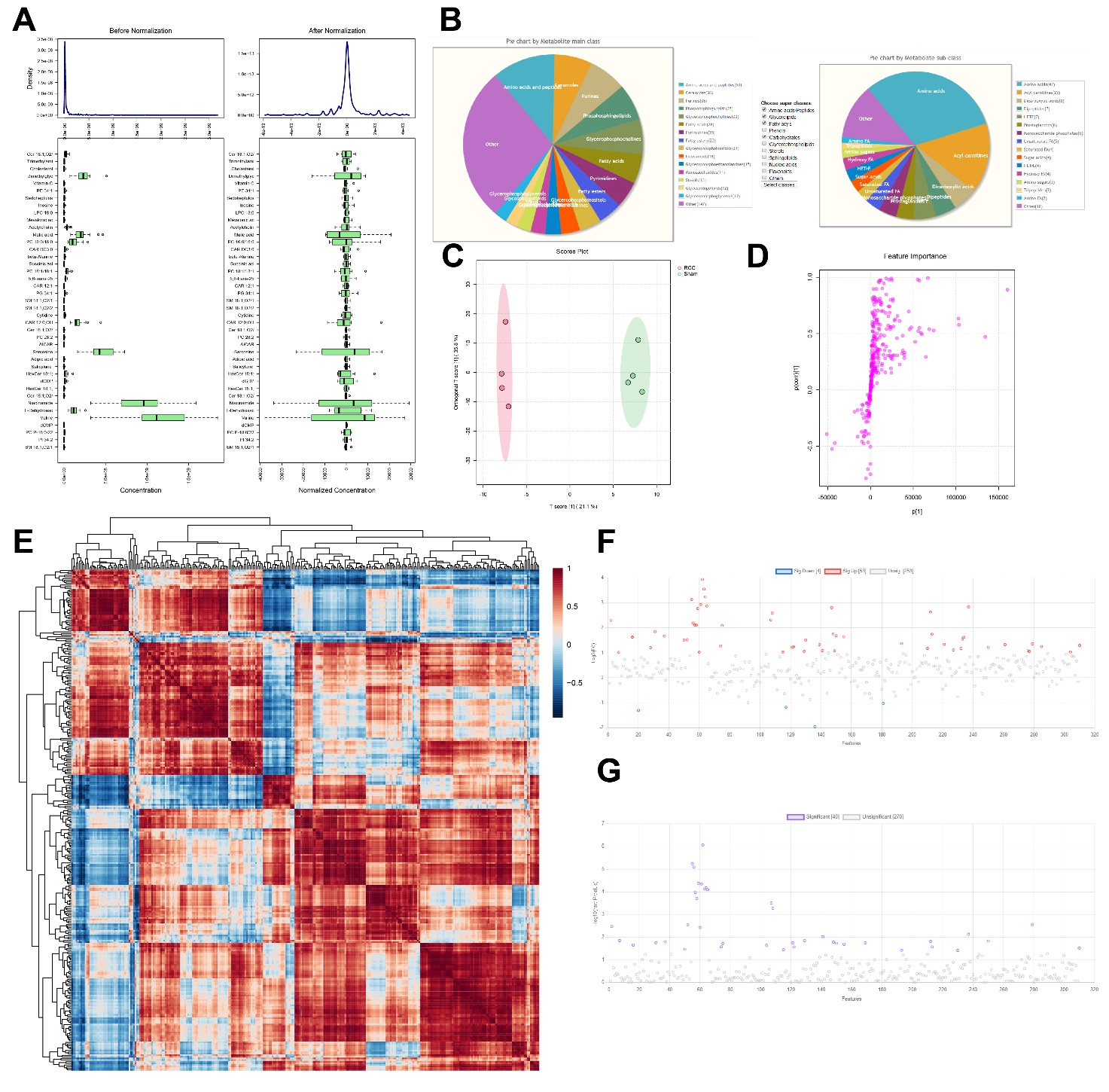


**Figure S2. Metabolomic data quality control and multivariate statistical analysis.**

Note: (A) Comparison of raw and normalized metabolomic data.; (B) Pie chart illustrating the composition of energy metabolism–related metabolites classified by “Main class” and “Sub class”; (C) OPLS-DA score plot displaying group separation along principal and orthogonal components; (D) S-plot of OPLS-DA analysis showing the importance of metabolites closer to the two angles of the plot; (E) Heatmap of correlation analysis of all metabolites; (F) Score plot of Fold Change analysis; (G) T-test analysis highlighting significantly different metabolites (red dots; *P* < 0.05). Each group included N=4 samples.


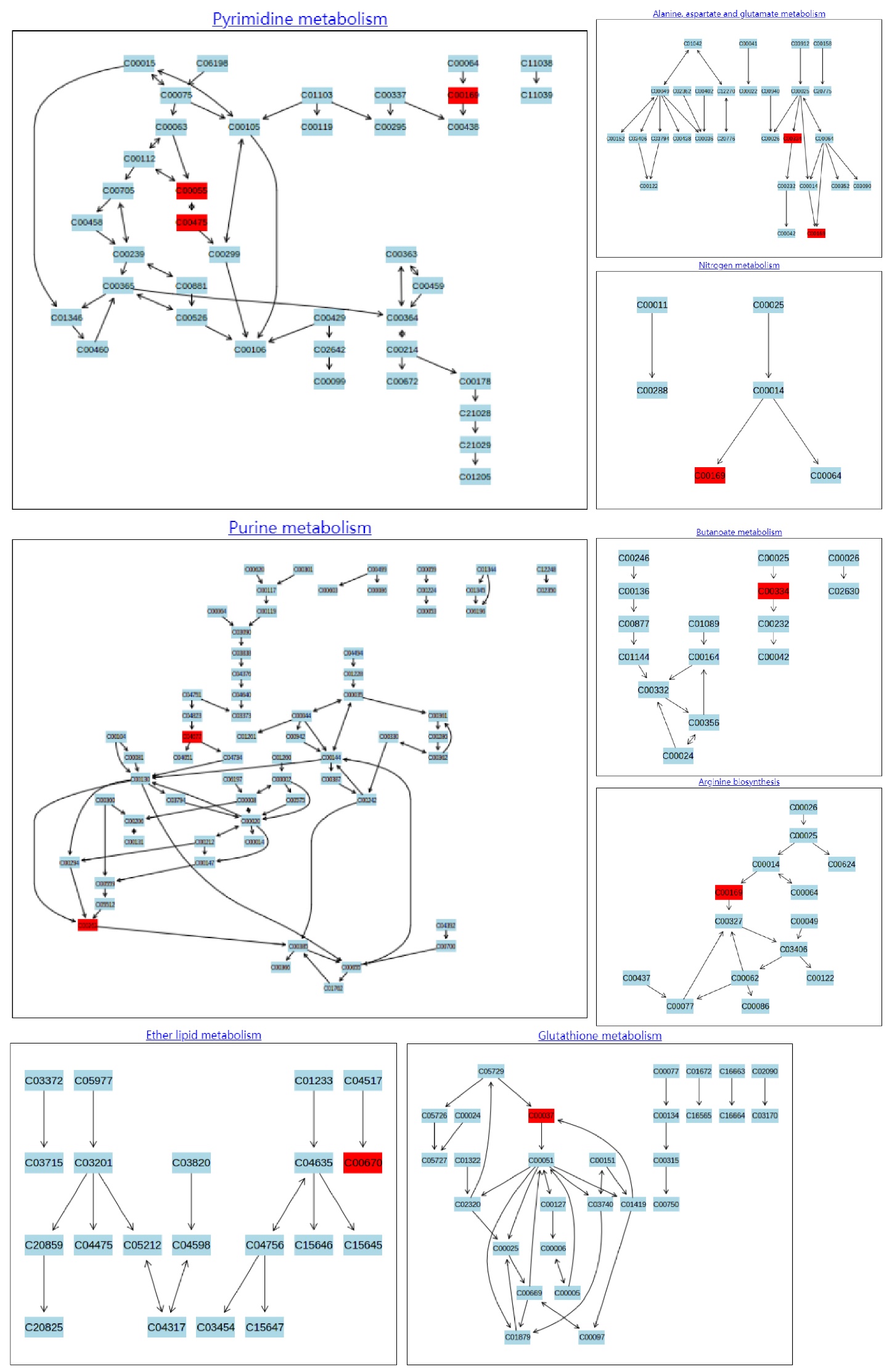


**Figure S3. Enrichment analysis results of differential metabolite pathways.**

Bar plot showing the top eight enriched pathways and associated metabolite categories based on functional analysis of differential metabolites (Created with BioRender.com).


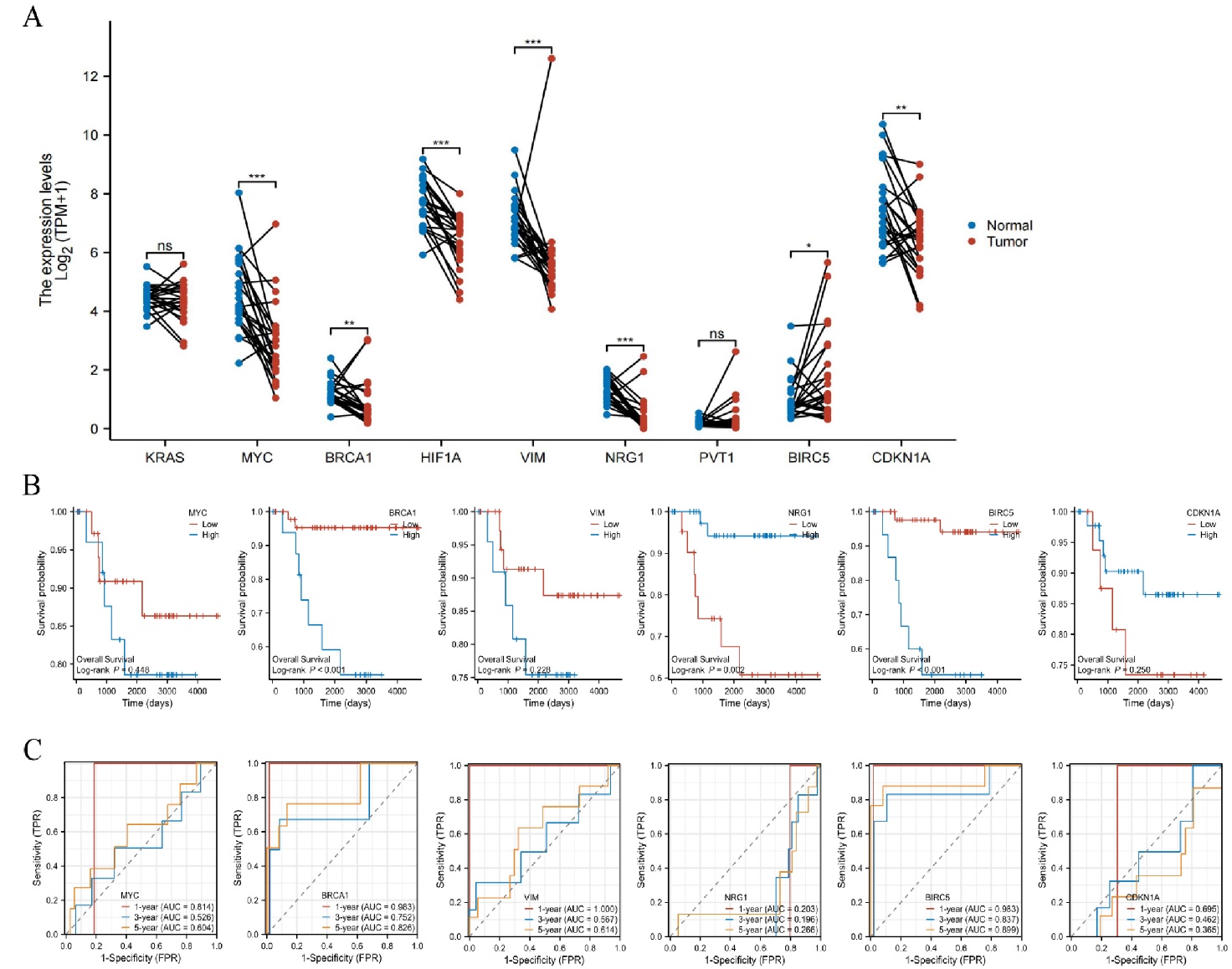


**Figure S4. Selection of prognosis-related factors from the TCGA database.**

Note: (A) Paired differential expression analysis of nine candidate genes, including KRAS and MYC, based on TCGA RCC data (N = 25 normal, N = 90 tumor samples). ns, *, and ** indicate no statistical difference, *P* < 0.05, and *P* < 0.01, respectively; (B) Survival analysis of seven differential genes such as BRCA1 and NRG1 using TCGA RCC cohort (N = 45 per group); (C) ROC curves for 1-, 3-, and 5-year survival prediction based on expression levels of these seven genes, with AUC values reflecting predictive accuracy.


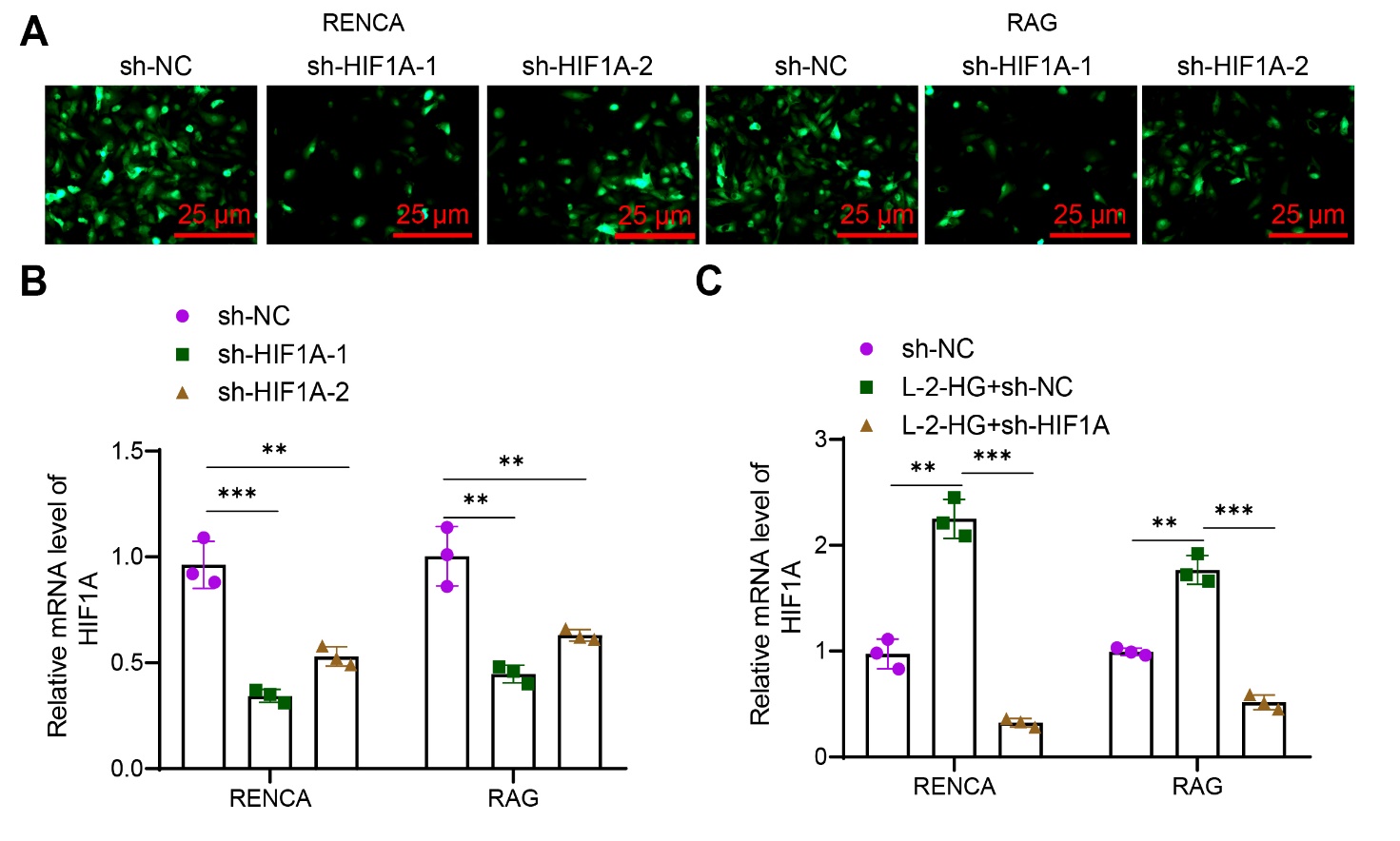


**Figure S5. Evaluation of cell transfection efficiency.**

Note: (A) Observation of cell transfection efficiency using fluorescence microscopy; (B) Detection of HIF1A expression levels in different transfection groups using RT-qPCR; (C) Detection of the impact of different treatments on HIF1A expression in cells using RT-qPCR. * and ** indicate *P* < 0.01 and *P* < 0.001, respectively, for the comparison between the two groups. The cell experiments were repeated three times.


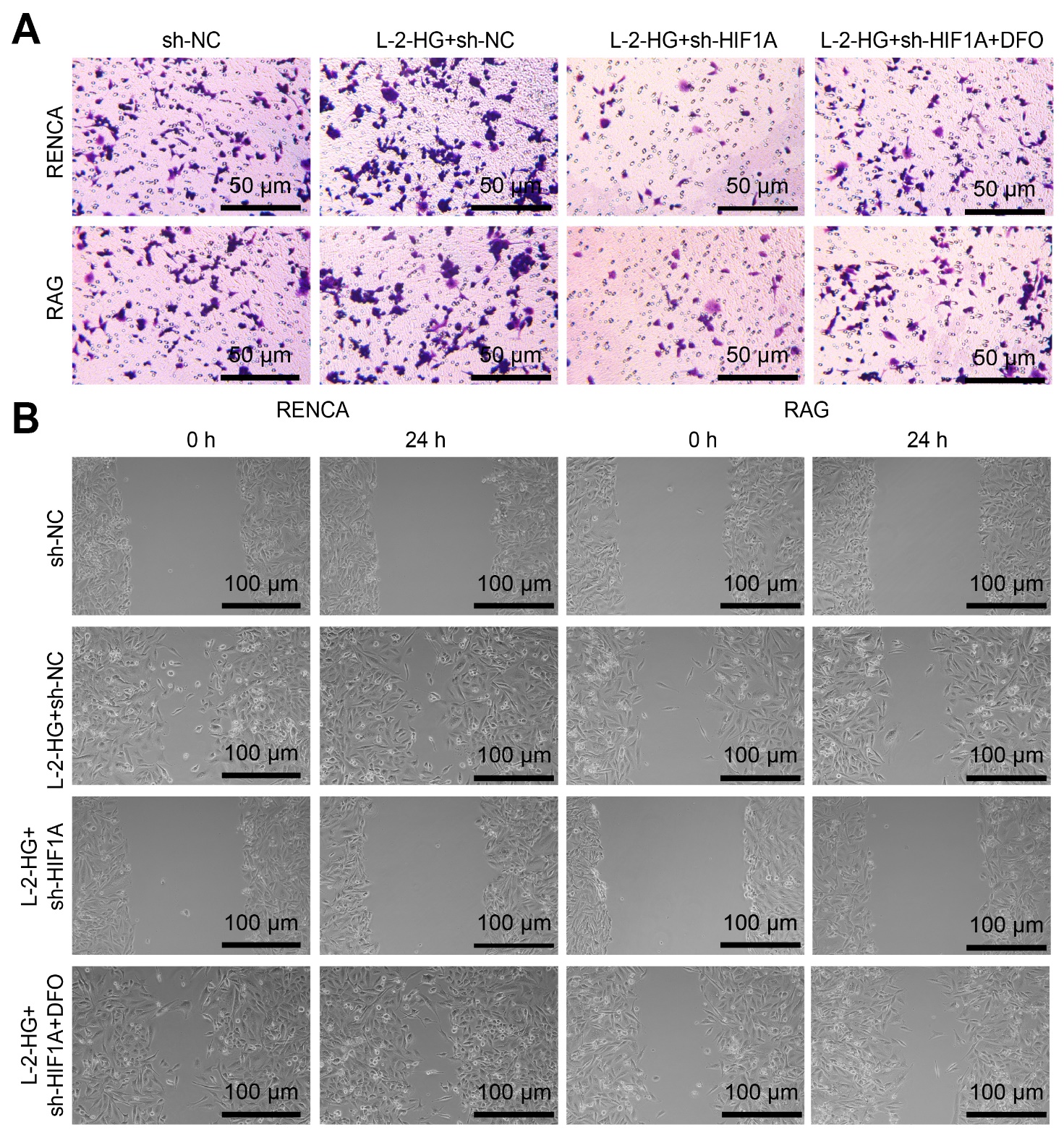


**Figure S6. Detection of malignant phenotypes in RCC cells.**

Note: (A) Transwell assay was conducted to detect the invasive ability of cells in each group; (B) Scratch assay was performed to detect the migration ability of cells in each group. The cell experiments were repeated three times.


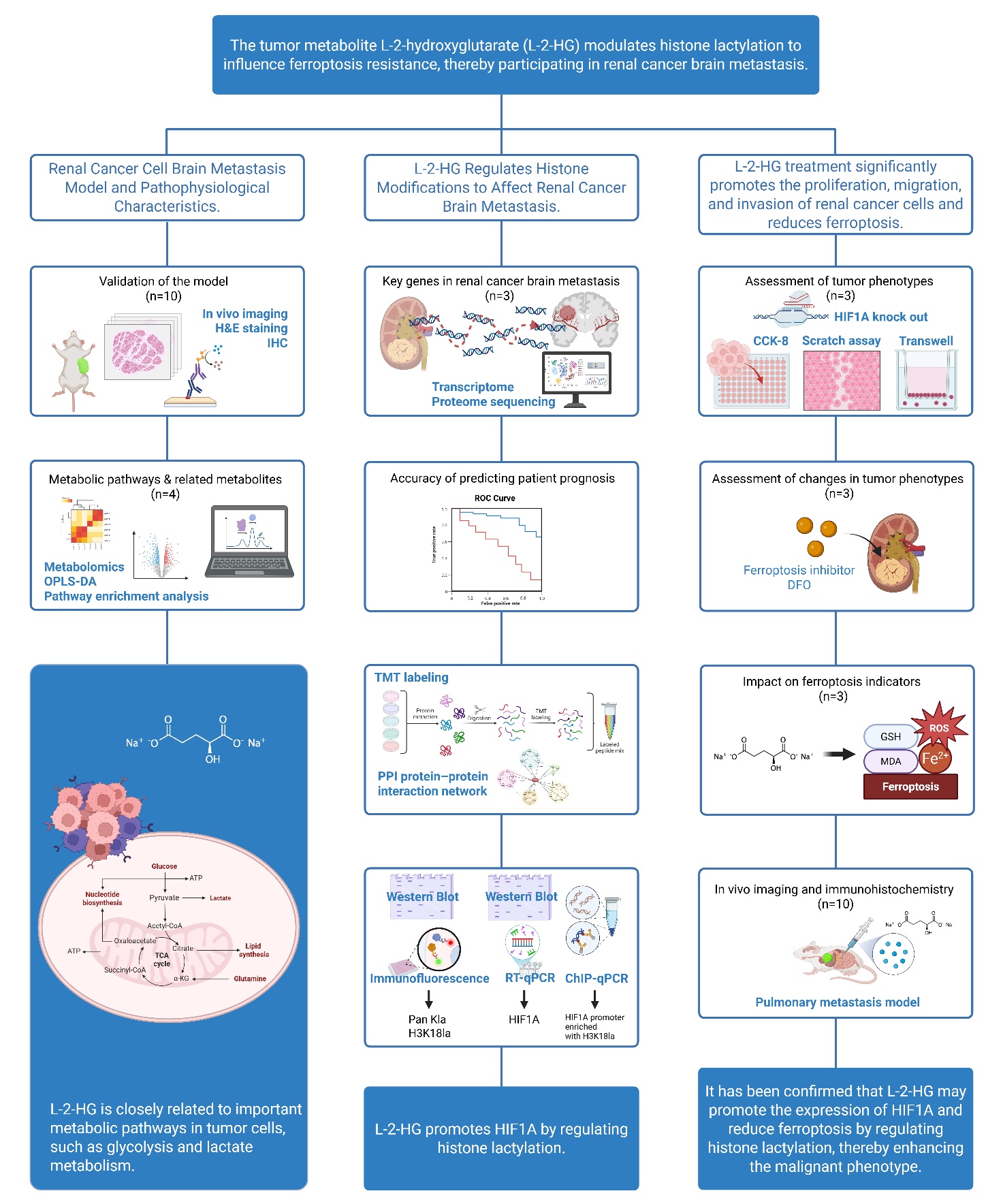


**Figure S7. Overview of experimental design, methods, and replicates.**Schematic illustration summarizing the major experimental workflows, including both *in vitro* and *in vivo* assays. Key abbreviations and the number of biological and technical replicates are presented for each method to aid in manuscript interpretation (Created with BioRender.com).


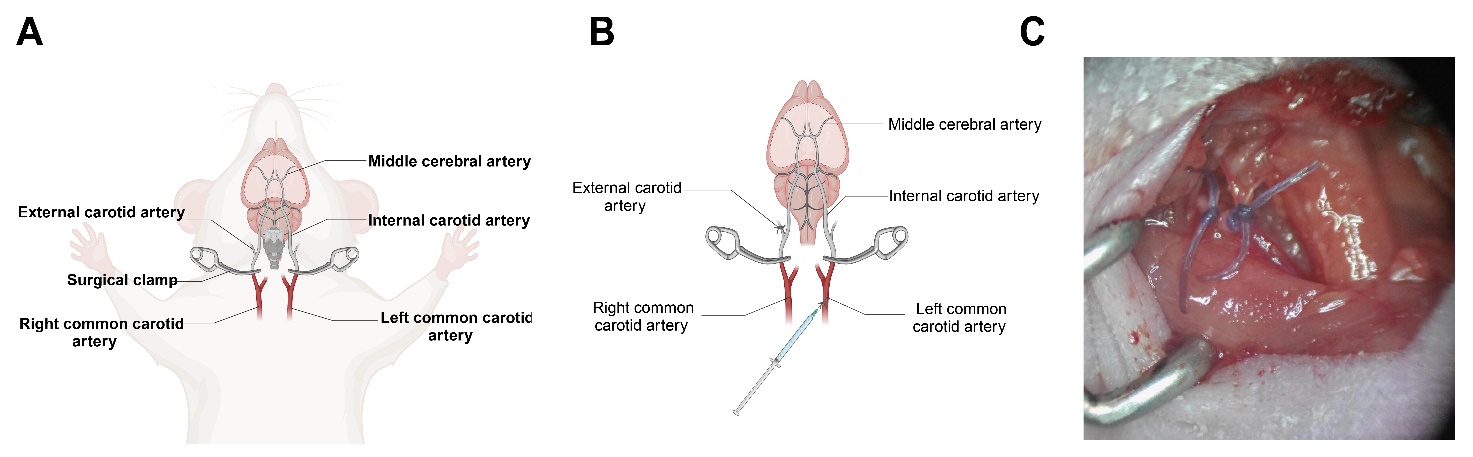


**Figure S8. Construction of mouse RCC brain metastasis model.**

Note: (A) Schematic diagram of mouse cranial anatomy (Created with BioRender.com); (B) Schematic diagram of tumor cell injection site and blood vessels (Created with BioRender.com); (C) Display of the carotid artery ligation experiment.
