# Supplementary material for: Novel role of L-2-HG in regulating HIF1A signaling pathway and iron death resistance in renal cancer brain metastasis
Source: Cell Death Dis. 2025 Nov 6;16(1):798. doi: 10.1038/s41419-025-08068-z (PMC12592421; doi:10.1038/s41419-025-08068-z)

Full and uncropped western blots of Figure 5A-1


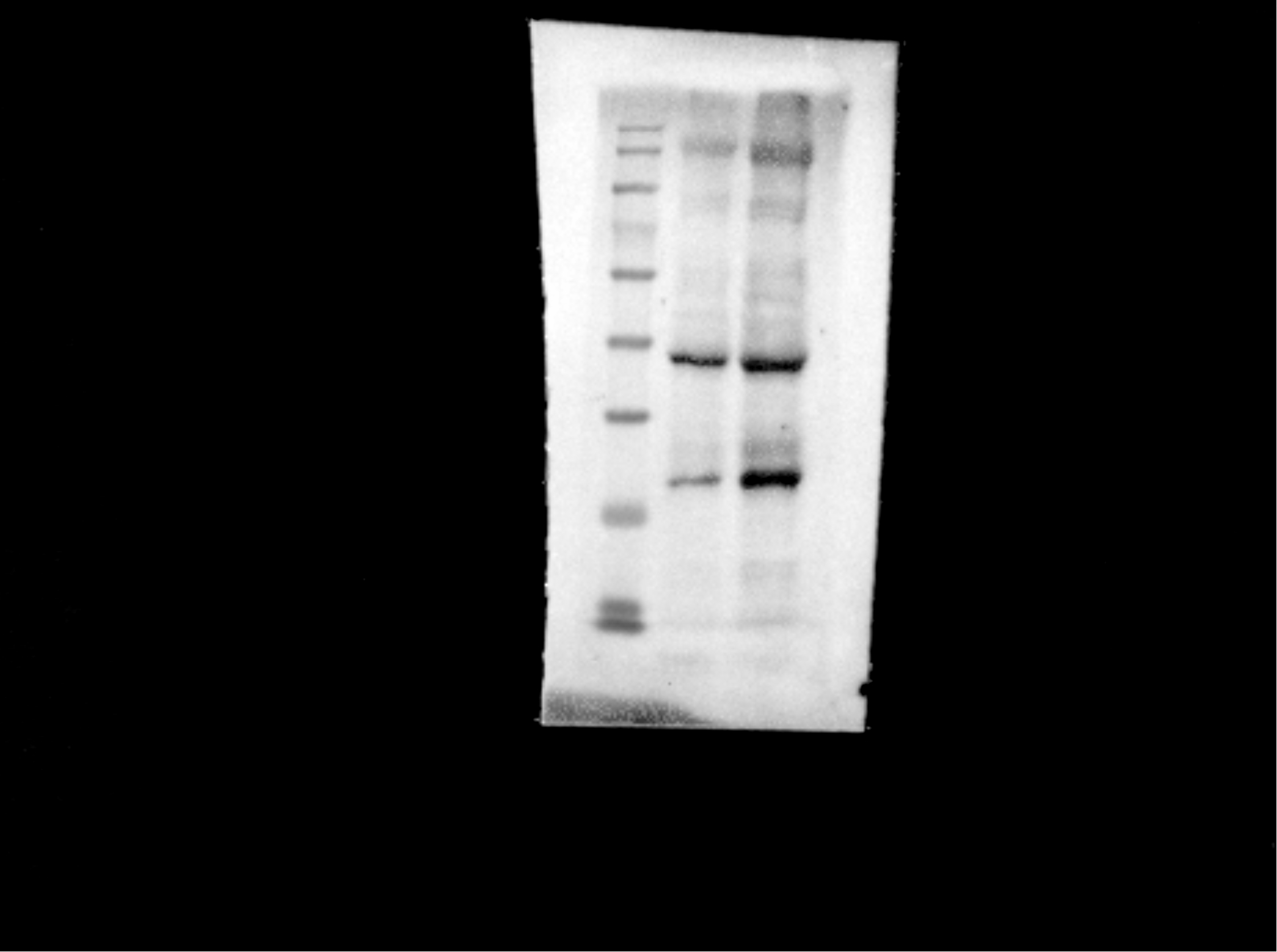


Full and uncropped western blots of Figure 5A-2


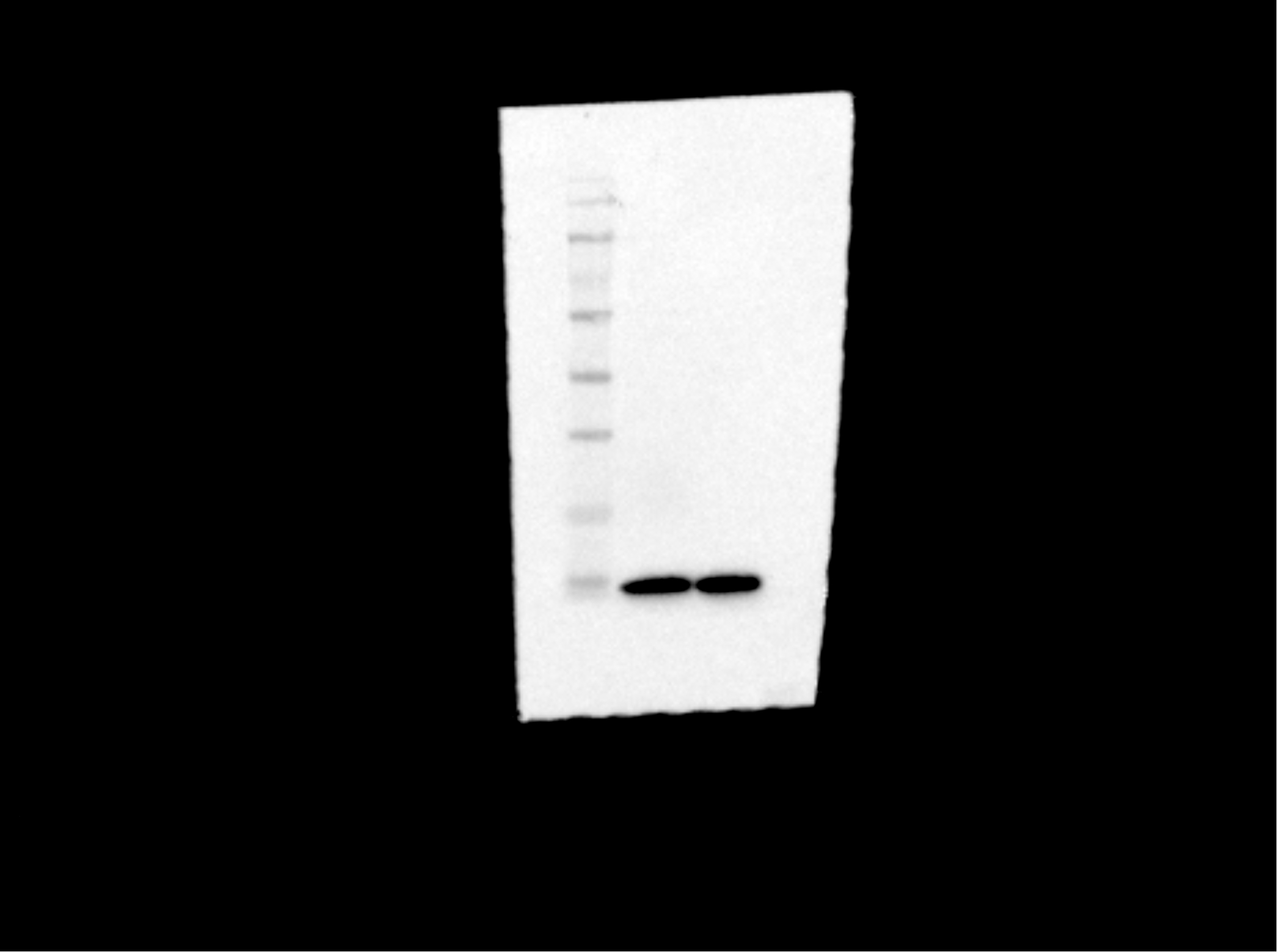


Full and uncropped western blots of Figure 5B-1


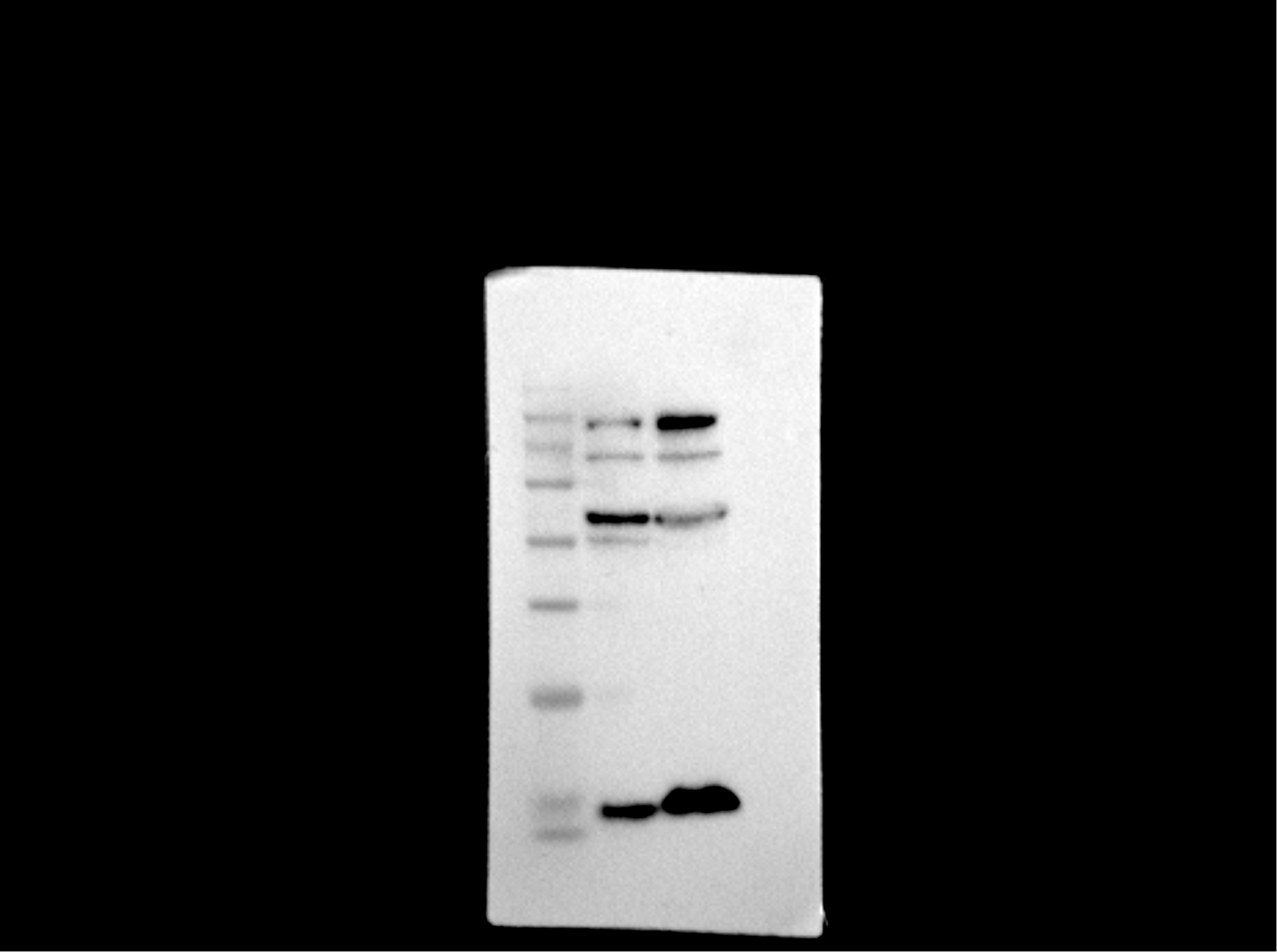


Full and uncropped western blots of Figure 5B-2


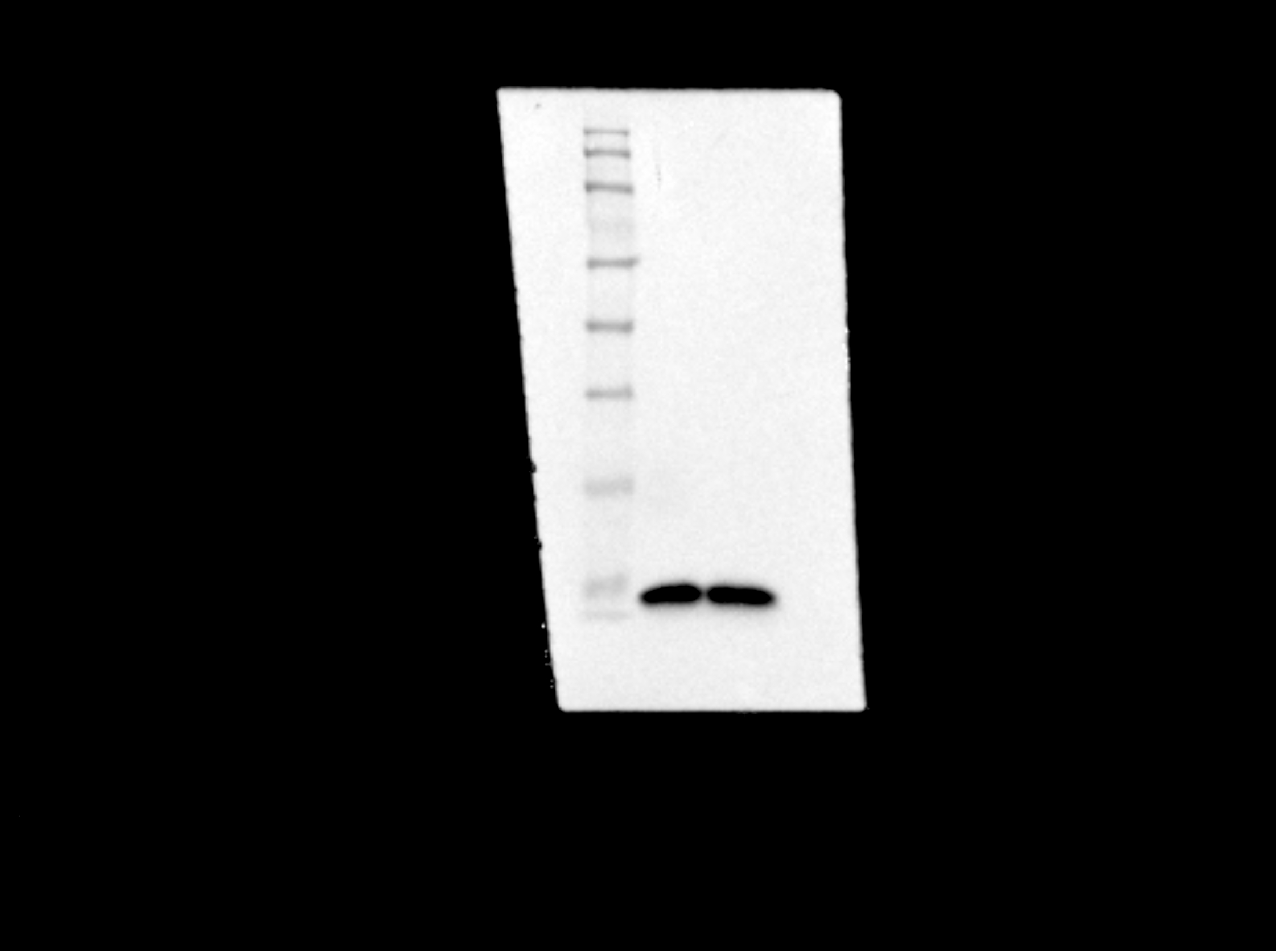


Full and uncropped western blots of Figure 5C-1


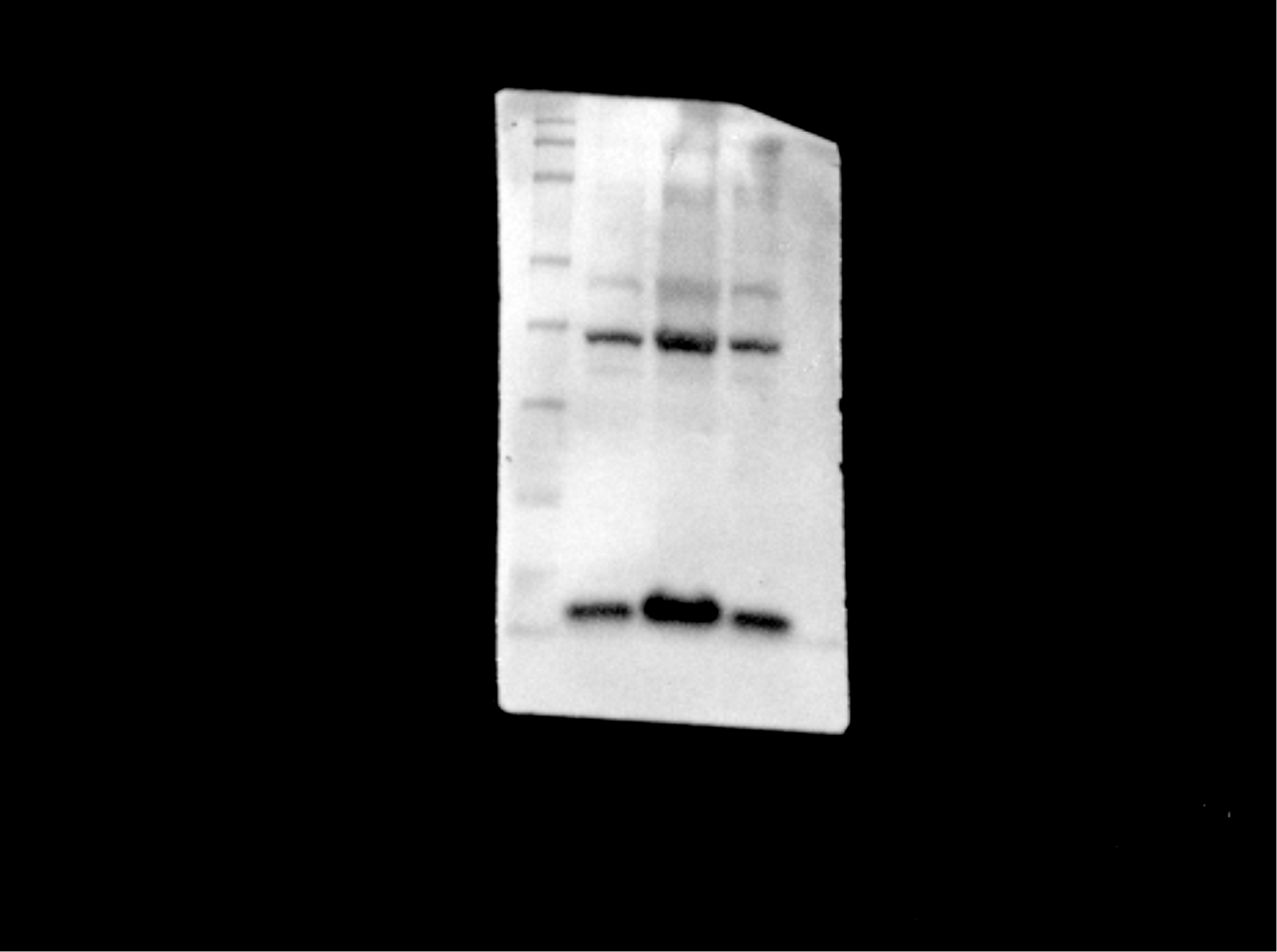


Full and uncropped western blots of Figure 5C-2


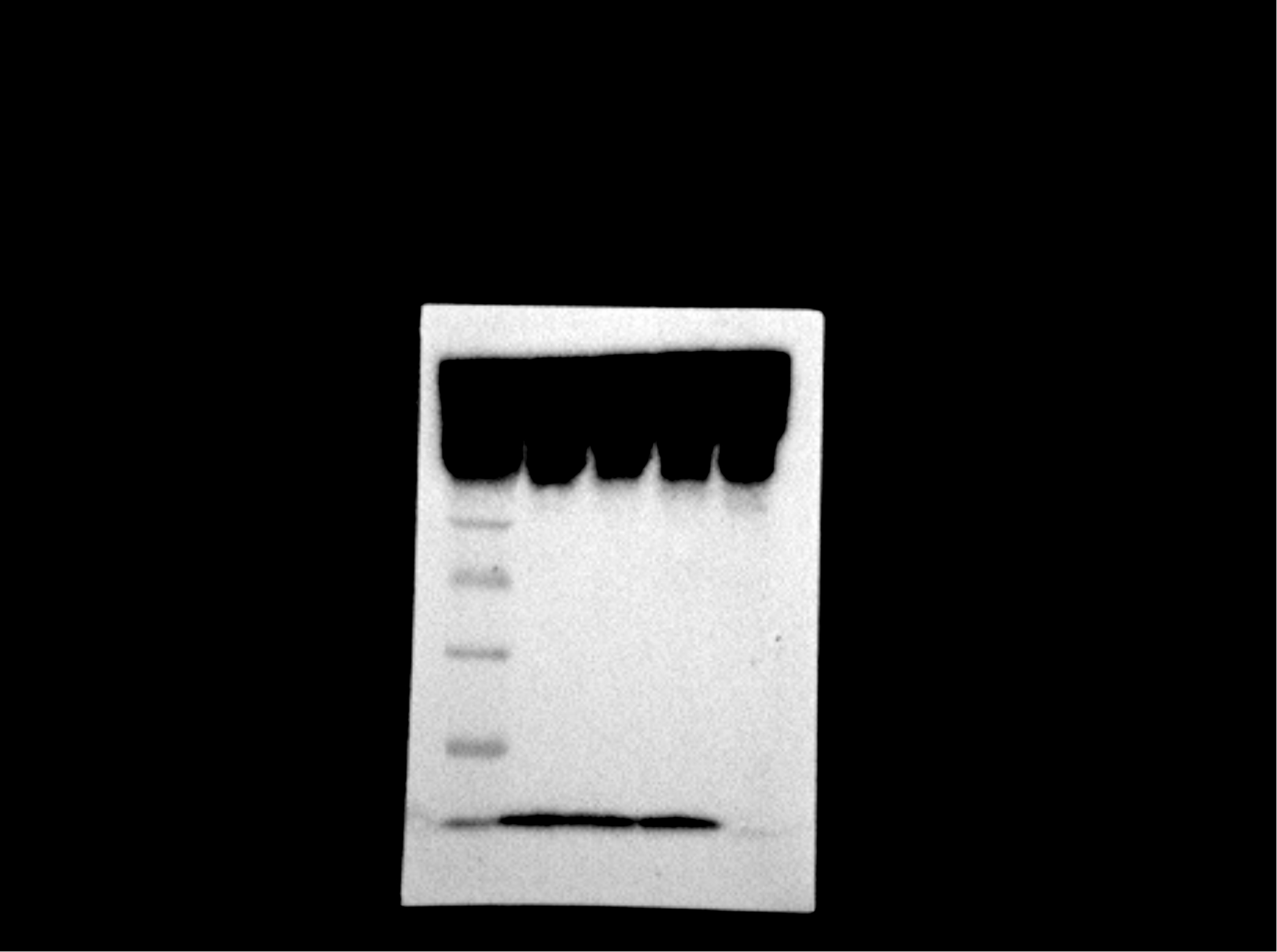


Full and uncropped western blots of Figure 5C-3


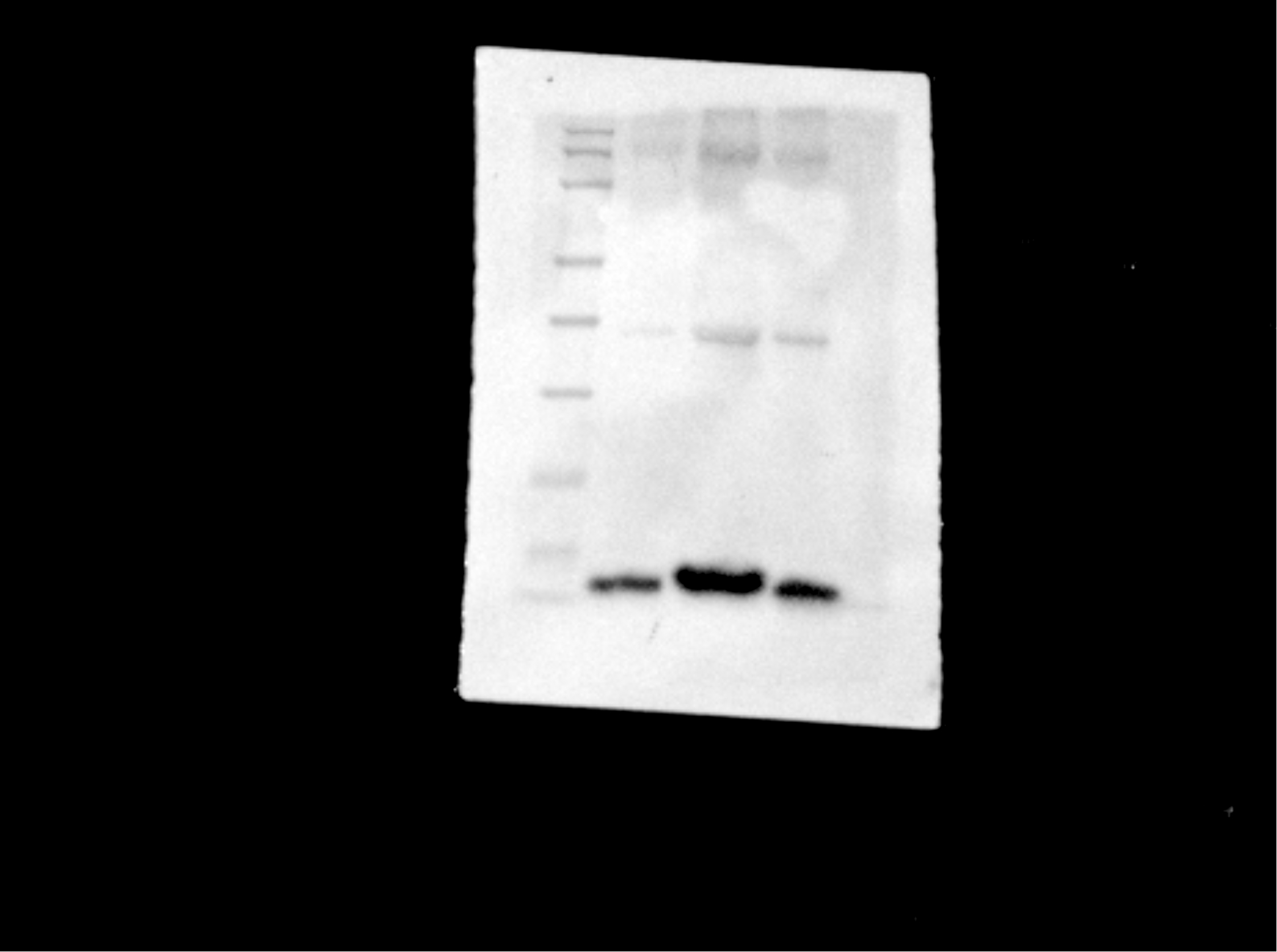


Full and uncropped western blots of Figure 5C-4


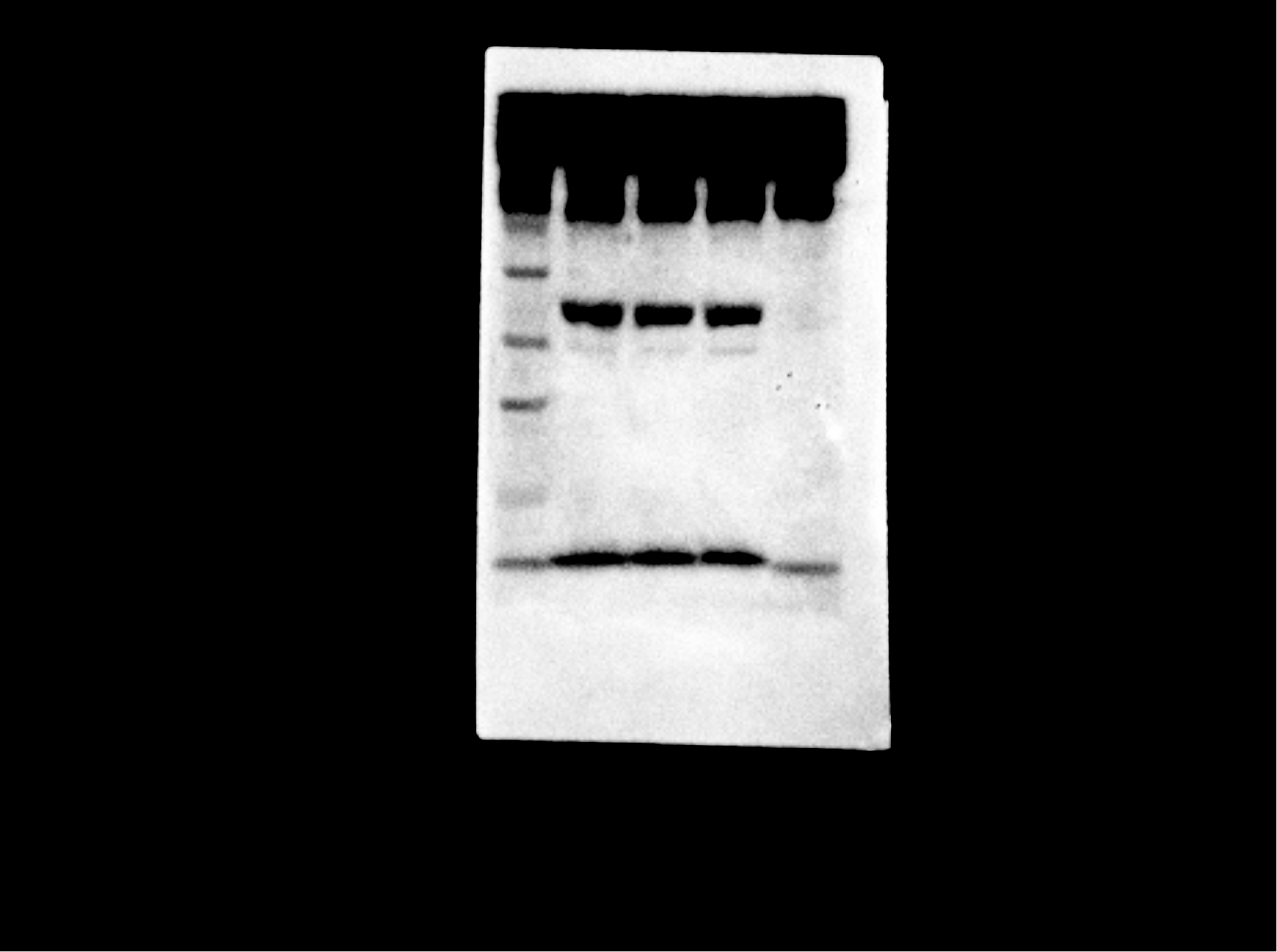


Full and uncropped western blots of Figure 5E-1


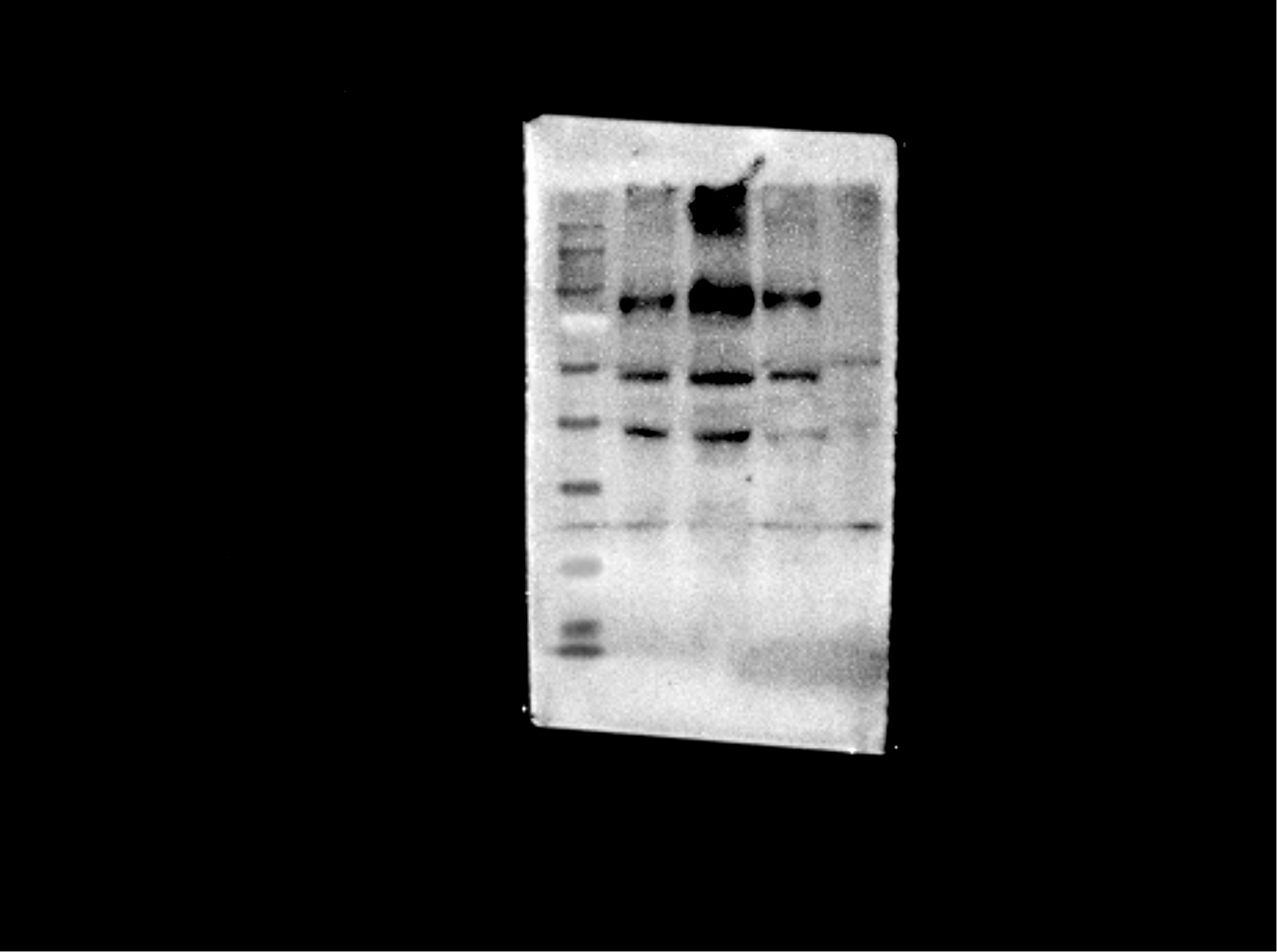


Full and uncropped western blots of Figure 5E-2


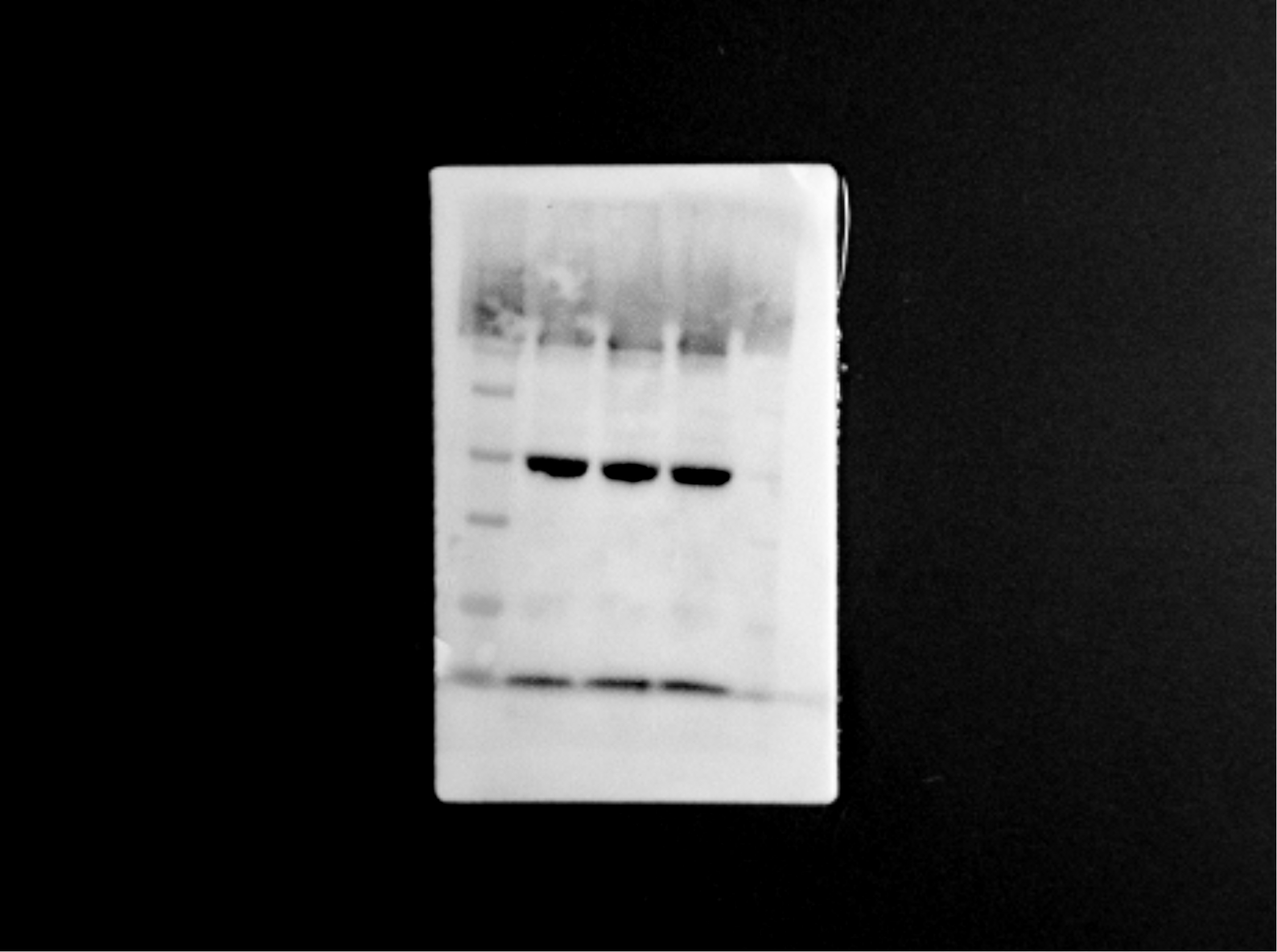


Full and uncropped western blots of Figure 5E-3


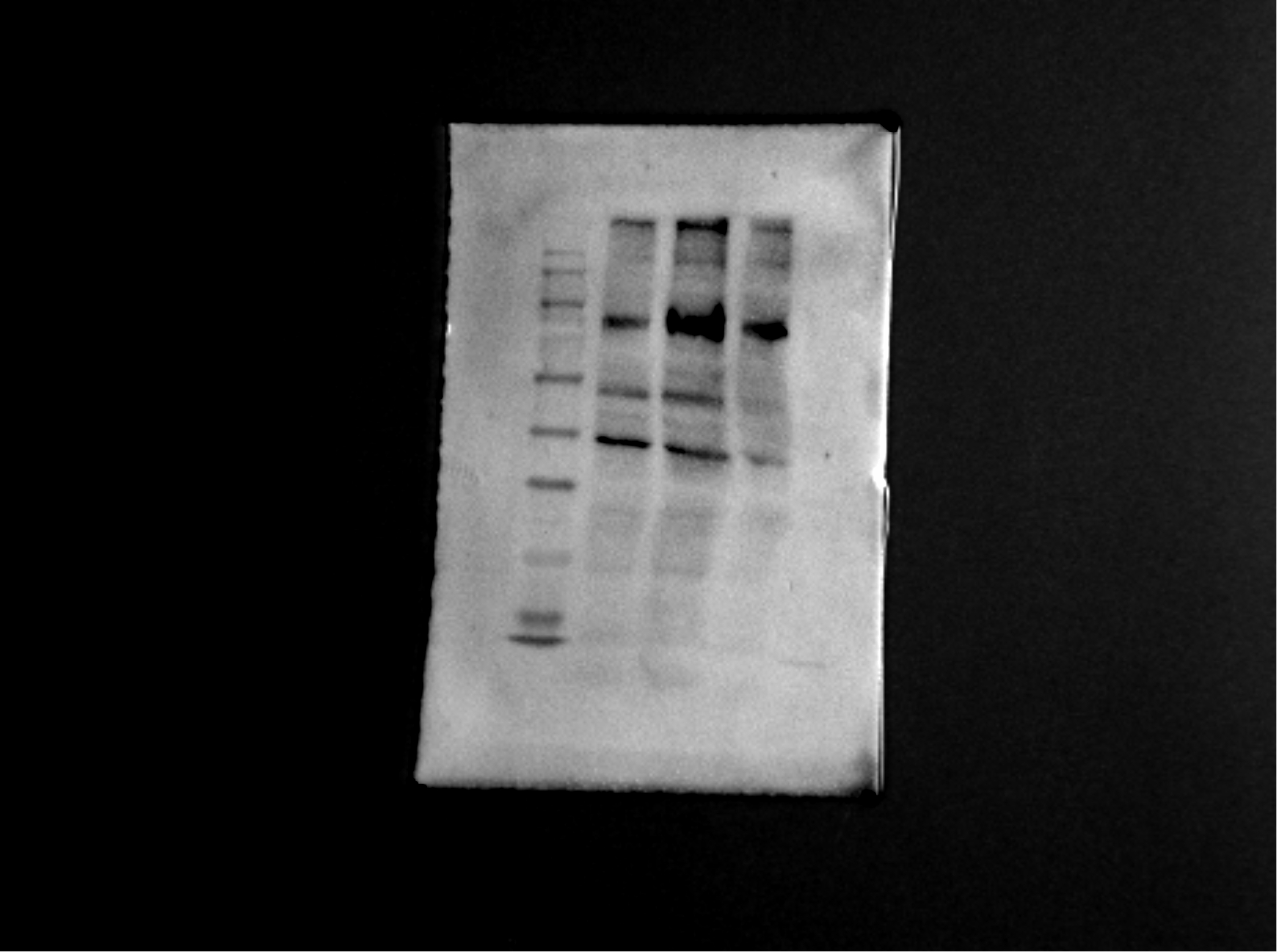


Full and uncropped western blots of Figure 5E-4


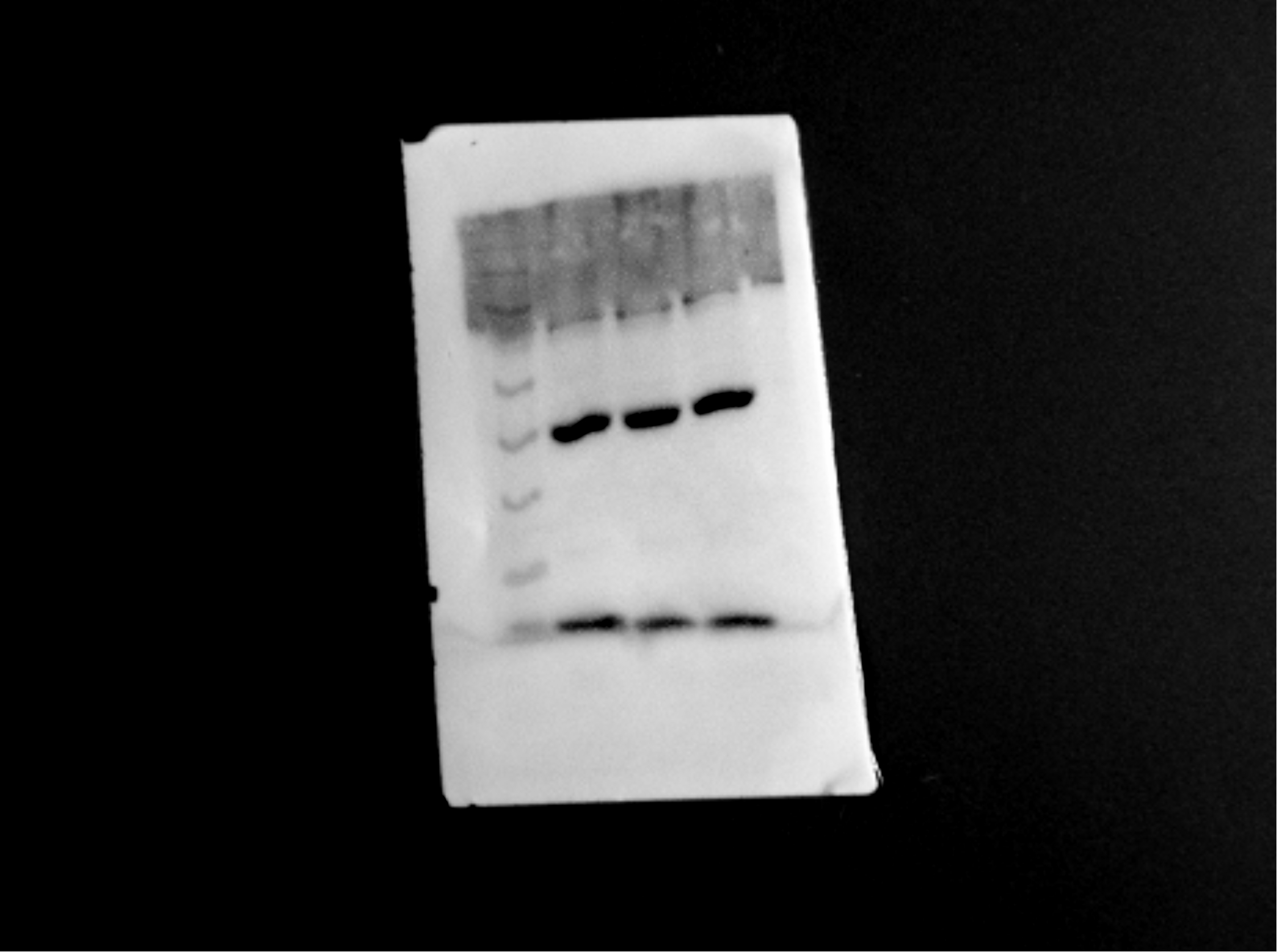

Supplement: Supplementary file 2 — Full and uncropped western blots [file 41419_2025_8068_MOESM2_ESM.docx]
